# High resolution age-structured mapping of childhood vaccination coverage in low and middle income countries

**DOI:** 10.1016/j.vaccine.2018.02.020

**Published:** 2018-03-14

**Authors:** C. Edson Utazi, Julia Thorley, Victor A. Alegana, Matthew J. Ferrari, Saki Takahashi, C. Jessica E. Metcalf, Justin Lessler, Andrew J. Tatem

**Affiliations:** a WorldPop, Department of Geography and Environment, University of Southampton, Southampton SO17 1BJ, UK; b Southampton Statistical Sciences Research Institute, University of Southampton, Southampton SO17 1BJ, UK; c Flowminder Foundation, Stockholm SE-11355, Sweden; d Center for Infectious Disease Dynamics, The Pennsylvania State University, State College, PA 16802, USA; e Department of Ecology and Evolutionary Biology, Princeton University, Princeton, NJ 08544, USA; f Department of Epidemiology, Johns Hopkins Bloomberg School of Public Health, Baltimore, MD 21205, USA

**Keywords:** Measles vaccine, Demographic and Health Surveys, Bayesian geostatistics, Coverage heterogeneities

## Abstract

**Background:**

The expansion of childhood vaccination programs in low and middle income countries has been a substantial public health success story. Indicators of the performance of intervention programmes such as coverage levels and numbers covered are typically measured through national statistics or at the scale of large regions due to survey design, administrative convenience or operational limitations. These mask heterogeneities and ‘coldspots’ of low coverage that may allow diseases to persist, even if overall coverage is high. Hence, to decrease inequities and accelerate progress towards disease elimination goals, fine-scale variation in coverage should be better characterized.

**Methods:**

Using measles as an example, cluster-level Demographic and Health Surveys (DHS) data were used to map vaccination coverage at 1 km spatial resolution in Cambodia, Mozambique and Nigeria for varying age-group categories of children under five years, using Bayesian geostatistical techniques built on a suite of publicly available geospatial covariates and implemented via Markov Chain Monte Carlo (MCMC) methods.

**Results:**

Measles vaccination coverage was found to be strongly predicted by just 4–5 covariates in geostatistical models, with remoteness consistently selected as a key variable. The output 1 × 1 km maps revealed significant heterogeneities within the three countries that were not captured using province-level summaries. Integration with population data showed that at the time of the surveys, few districts attained the 80% coverage, that is one component of the WHO Global Vaccine Action Plan 2020 targets.

**Conclusion:**

The elimination of vaccine-preventable diseases requires a strong evidence base to guide strategies and inform efficient use of limited resources. The approaches outlined here provide a route to moving beyond large area summaries of vaccination coverage that mask epidemiologically-important heterogeneities to detailed maps that capture subnational vulnerabilities. The output datasets are built on open data and methods, and in flexible format that can be aggregated to more operationally-relevant administrative unit levels.

## Highlights

• Geostatistical models showing strong predictive performance are used to produce maps of measles vaccination coverage at 1 × 1 km resolution.
• Remoteness, measured as travel time to nearest major settlement, was consistently a key predictor of coverage.
• The maps reveal heterogeneities and ‘coldspots’ of low vaccination coverage that are missed using large area summaries.
• Aggregated estimates of coverage that do not account for local heterogeneities potentially over-estimate the numbers of children vaccinated by over 10%.
• Relating to the WHO GVAP targets of 80% coverage, the integration of high resolution coverage and population maps shows the districts that have attained the threshold in the study countries.

## 1 Introduction

Health policy decision-making based on spatially heterogeneous vaccination has resulted in a shift from pursuing coverage targets at the national-level to ensuring that high coverage levels are evenly distributed across provinces or districts [1]. While this likely represents a more effective strategy over targeting country-level goals, administrative area summaries may still mask important geographical inequities in coverage [2]. Small regions of susceptibility formed by spatial clustering of unvaccinated individuals can sustain disease transmission, even when high overall vaccination coverage is achieved. Continued disease circulation can also be driven by age cohorts that are missed by routine vaccination, unless they are removed from the susceptible population through natural infection or vaccination campaigns that target broader age ranges [3], [4].

To better capture heterogeneities in vaccine coverage, two general options exist, either increasing the intensity of surveys or using statistical modelling approaches. The former is costly, and therefore modelling approaches that leverage existing survey data, spatial relationships between survey clusters and relationships with geospatial covariates have become increasingly popular in mapping key development indicators at high spatial resolution. Driven by rapid increases in computing power, rising availability of a range of detailed geospatial datasets and advances in statistical methods, recent examples include the mapping of age structures [5], poverty [6], malaria prevalence [7], sanitation [8] and literacy [9] within Bayesian geostatistical frameworks that enable quantification and mapping of uncertainty in estimates. These efforts have revealed new insights into the spatial heterogeneities of health and development metrics, as well as producing more precise estimates of populations at risk or affected when combined with high resolution population maps (e.g. [10]).

Here we explore the potential of geostatistical approaches to modelling age-structured vaccination coverage across three countries, using measles vaccine as an example. Geolocated cluster survey data are combined with a library of candidate geospatial layers capturing covariates such as urbanicity, remoteness and poverty, to test their ability to predict vaccination coverage at high spatial resolution and estimate numbers covered when combined with population maps.

## 2 Methods

**Fig. 1:**
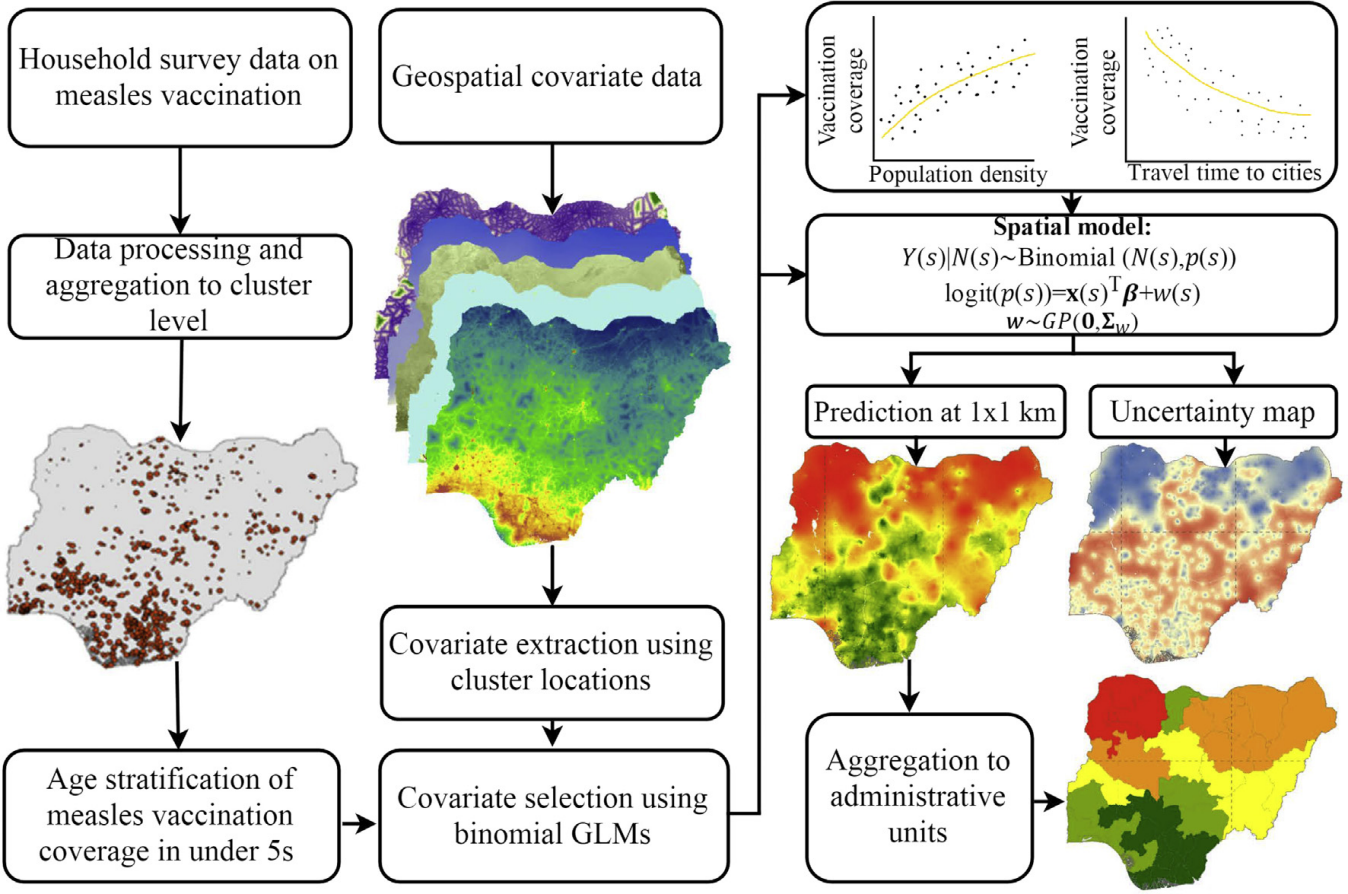
A schematic diagram of the modelling approach used to produce high resolution age-structured estimates of vaccination coverage.

Fig. 1 depicts an overview of the modelling approach used in this work from data assembly to model outputs, using Nigeria as an example. Each stage is described in the following sections, and in greater detail in supplemental materials.

### 2.1 Measles vaccination coverage data

The Demographic and Health Surveys (DHS) program conducts nationally representative household surveys that provide data on a wide range of demographic and health indicators in low and middle income countries [11]. Cross-sectional data on the spatial distribution of measles vaccination coverage in children under 5 years of age for Cambodia, Nigeria and Mozambique were obtained from the DHS database [12]. For each child surveyed, the measles vaccination status, i.e. whether they had ever received a measles vaccine or not, as determined from the vaccination card or as reported by the mother, was extracted. In this definition of measles vaccination coverage, used by the Demographic and Health Surveys program [12], [13], [14], there is an implicit assumption that the child has at least received the first dose of measles containing vaccine (MCV-1), but could also have had the second dose (MCV-2). Other information obtained included the child’s age in months at the time of the survey and the centroid of the cluster from which the child’s household was selected. To maintain confidentiality, DHS cluster centroids are randomly displaced up to 2 km in urban areas and 5 km in rural areas [15], and this displacement was accounted for during covariate data extraction following recommended approaches [16]. For each country, only the most recent survey was used, corresponding to 2014, 2011 and 2013 for Cambodia, Mozambique and Nigeria, respectively. Overall, 22,897 children in these countries were vaccinated against measles out of a total of 45,297 children with complete records. Fig. S1 (supplemental material) maps the cluster locations and the proportions of under-5 s vaccinated in each country. For a variety of reasons, vaccination coverage is often evaluated by age [2], [17], [18]. In this work, we defined four age intervals relevant to coverage assessments: <9 months, 9–11 months, 12–23 months and 24–59 months, and also analysed coverage in the under 5 year age category (i.e. <59 months).

### 2.2 Covariate data, processing and selection

Many geospatial socio-economic, environmental and physical factors are known to directly or indirectly influence or be associated with the spatial distribution of the under-five demographic and geographical inequities in vaccination coverage [5], [19], [20], [21]. In a spatial regression context, these covariate factors can aid in explaining the observed spatial distributions of measles vaccination coverage and are also particularly important for prediction. For this analysis, we assembled a suite of candidate covariate data layers that are documented in Table S1 (supplemental material), and include factors such as remoteness (measured as travel time to nearest large settlement) and related infrastructure metrics that have been shown to be associated with vaccination coverage [21], [22]. Additional categories of covariates include demographics, as vaccination coverage can vary by population density and ethnic groups [23], [24], and economic metrics, since coverage has been shown to vary with rates of poverty [25], [26]. A set of land use/cover, topographic and climate/environment variables were also tested for their ability to predict coverage rates. While these may not have a direct link to coverage, they are often associated with social, health, access and demographic factors that underlie geographic variations, and have been shown to be associated with demographics, wealth and various other development indicators [5], [6], [8], [9]. The values of each covariate dataset at the locations of the DHS clusters were extracted. Examples of the covariate data, the processing details and the covariate selection step which was performed using non-spatial binomial generalized linear models (GLMs) in a frequentist framework are provided in supplemental materials.

### 2.3 Model fitting and validation

For each of the age groups defined previously, we model $Y(s_{i})$, the number of children vaccinated at cluster location $s_{i}(i=1\text{,}\ldots \text{,}n)$, using a binomial spatial regression model (see, e.g. [27]), with probability of success, $p(s_{i})$. Letting $N(s_{i})$ denote the total number of children surveyed at cluster $s_{i}$, the model can be written as:

(1) $$\begin{array}{cc} & Y(s_{i})|N(s_{i})∼\text{Binomial}(N(s_{i})\text{,}p(s_{i}))\text{,}\\ & \text{logit}(p(s_{i}))=x{\left(s_{i}\right)}^{\text{T}}\beta +w(s_{i})\text{,}\\ & w(s_{1})\text{,}\ldots \text{,}w(s_{n})∼\mathrm{GP}(0\text{,}\Sigma_{w})\text{,} \end{array}$$

where $x(s_{i})$ is a set of covariates associated with cluster $s_{i}$ and β are the corresponding regression parameters. $w={\left(w(s_{1})\text{,}\ldots \text{,}w(s_{n})\right)}^{T}$ is a zero-mean stationary Gaussian Process with covariance matrix, $\Sigma_{w}$, used to model spatial dependence in the data. The random effects w are also used to capture the effect of spatially-varying covariate factors that are not included in the model. A popular specification for $\Sigma_{w}$ from the Matérn family of covariance functions [28] used in this work is the exponential function given by $\Sigma_{w}=\sigma^{2}\text{exp}(-\phi D)$, where $\sigma^{2}>0$ and ϕ>0 are known as the partial sill and the spatial decay parameters, respectively, and D is a matrix of known Euclidean distances between the cluster locations.

The geostatistical model in (1) was fitted in a Bayesian framework using MCMC methods (see supplemental materials for details). Using the fitted model, we predicted the age-specific probabilities, p(s), of being vaccinated at 1 × 1 km resolution for each country. The predicted probabilities were then aggregated to policy-relevant administrative areas for each country. The coefficients of determination (R^2^) of the fitted models were used to evaluate their predictive power. Further, to assess the performance of the models for out-of-sample prediction, a cross-validation exercise was carried out in each case. Percentage bias, validation mean square error and nominal coverage of the 95% prediction intervals, all of which are described in the supplemental materials, were used to quantify predictive performance. The model was implemented using the spBayes package in R [29], [30].

## 3 Results

### 3.1 Covariate selection and model construction

The selected covariates for modelling and predicting vaccination coverage for Cambodia were travel time, population density, distance to residential areas and distance to infrastructures. For Mozambique, these were: travel time, precipitation, evapotranspiration and net primary production. For Nigeria, travel time, poverty, aridity, night-time light intensity and enhanced vegetation index were selected for the analysis. The results highlight that remoteness (measured as travel time to major settlements) is an important predictor of vaccination coverage having been selected in all the countries tested, as well as matching previous findings [21].

The selected covariates were used in the spatial model described previously to model and predict the probability of being vaccinated against measles at 1 × 1 km spatial resolution for each age cohort in the three countries studied at the time of their DHS survey. The estimates of the parameters of the fitted models including the regression coefficients are reported in Table 1 for the 0–59 month age group and for other age groups in supplemental materials (Tables S2–S4).

**Table 1:** Estimates of the parameters of the fitted models for age 0–59 months. Reported are the posterior means, standard deviations and quantiles (2.5%, 50% and 97.5%) of the regression coefficients and the parameters of the spatial random effect, w.

Parameter	Mean	SD	2.5%	50%	97.5%
*Cambodia*					
(Intercept)	0.8646	0.7889	−0.7442	0.8895	2.2519
log (travel time)	−0.0225	0.0571	−0.1324	−0.0221	0.0962
log (population density)	0.0953	0.0427	0.0096	0.0961	0.1773
log (distance to residential areas)	0.0153	0.0467	−0.0829	0.0180	0.1023
log (distance to infrastructures)	−0.0499	0.0441	−0.1386	−0.0478	0.0359
Partial sill ($\sigma^{2}$)	0.1903	0.0363	0.1288	0.1874	0.2701
Spatial decay (ϕ)^a^	30.7648	17.2687	7.7638	26.6455	67.1314

*Mozambique*					
(Intercept)	3.3841	0.6232	1.9759	3.4159	4.5262
log (travel time)	−0.0558	0.0340	−0.1202	−0.0569	0.0109
Precipitation	−0.0095	0.0021	−0.0134	−0.0096	−0.0051
Evapotranspiration	−0.0009	0.0004	−0.0016	−0.0009	−0.0001
log (net primary production)	0.2486	0.1993	−0.1518	0.2517	0.6395
Partial sill ($\sigma^{2}$)	0.3061	0.0399	0.2351	0.3037	0.3903
Spatial decay (ϕ)^a^	17.5505	11.7127	4.7154	14.4912	52.3511

*Nigeria*					
(Intercept)	−1.1970	0.4300	−1.9973	−1.1915	−0.2504
Poverty	−0.9333	0.4834	−1.8746	−0.9455	0.0392
Aridity	0.0001	2.93 × 10^−5^	6.68 × 10^−5^	0.0001	0.0002
log(Night-time lights)	0.3840	0.0753	0.2408	0.3844	0.5329
log(travel time)	−0.1438	0.0519	−0.2378	−0.1463	−0.0417
EVI	2.5418	0.7748	1.0493	2.5170	4.1947
Partial sill ($\sigma^{2}$)	1.8089	0.2590	1.3942	1.7784	2.4145
Spatial decay (ϕ)^a^	1.9417	0.3267	1.3237	1.9331	2.6197

a These correspond to effective ranges of 13 km, 23 km and 173 km, respectively (see supplemental materials for details).

We note that the covariates are measured on differing scales, and therefore, the estimated coefficients of the covariates were not directly comparable. Covariates for which the coefficients had 95% credible intervals, i.e. the intervals formed by the 2.5% and 97.5% quantiles, that did not include zero were identified as having strong/consistent associations with vaccination coverage. The estimates showed that vaccination coverage generally decreased with increases in remoteness (except in the 0–8 month age group where parameter estimates have been biased relative to other age groups due to insufficient data), with this association being consistent across most age groups in these countries. In Cambodia, the probability of being vaccinated generally increased with increasing population density (consistent in 0–59 month and >12 month age groups) and decreased with increasing distance to infrastructure and residential areas (both consistent in the 9–11 month age group). In Nigeria, an increase in poverty was associated with a reduction in vaccination coverage whereas aridity, night-time light intensity and vegetation amount were each positively correlated with vaccination coverage. Additionally, the estimated associations between all the covariates and vaccination coverage were found to be consistent for age groups 24–59 months and 0–59 months. For Mozambique, precipitation and evapotranspiration were both consistently negatively correlated with vaccination coverage in the 0–59 month age group. Also, a strong positive association was found between net primary production and vaccination coverage in the 24–59 month and 0–59 month age groups in this country.

The 95% credible intervals of the parameters of the spatial random effect, w, did not include zero; thus confirming the presence of significant spatial dependence in the data. The estimates of the spatial correlation decay parameter, ϕ, suggest the presence of local residual spatial correlation in the models for Cambodia and Mozambique (effective spatial range ⩽71km, i.e. a distance at which spatial dependence is negligible – see supplemental materials for details) across all the age groups. For Nigeria, the same pattern was seen in the lower age groups, but relatively higher levels of spatial correlation were estimated in age groups ⩾ 12 months, with effective ranges of up to 286km.

### 3.2 Model validation

Model validation statistics showed that for Nigeria, the nominal coverage of the 95% prediction intervals ranged between 92.78% and 95.63%, which indicate good approximations of the true value. For Cambodia and Mozambique, the coverage values were at least 91% in all cases. Although for the lower age groups in Cambodia (<12 months), the values obtained were too high; this was most likely as a result of high uncertainties arising from small sample sizes at the cluster locations. Percentage bias was generally low for all countries and age groups, ranging between −2.84% and 2.80%, and extending up to −8.20% only in the 0–8 month age group. As expected, due to increasing amounts of data being available for model-fitting, better predictions were obtained for the 0–59 month age group and other age groups greater than 11 months, as mean square error values also revealed. R^2^ values generally indicated a strong predictive power in the fitted models for the combined 0–59 month age group, with all values being >0.65, and as high as 0.95 for Nigeria. For the age-structured models, with the exception of a few cases (ages 0–8 and 9–11 months in Cambodia; 9–11 months in Mozambique), the covariates used in these models were shown to explain at least 50% of the variation in the observed probabilities of being vaccinated. The full model validation statistics for all countries are reported in supplemental materials (Table S5).

### 3.3 Vaccination coverage maps

**Fig. 2:**
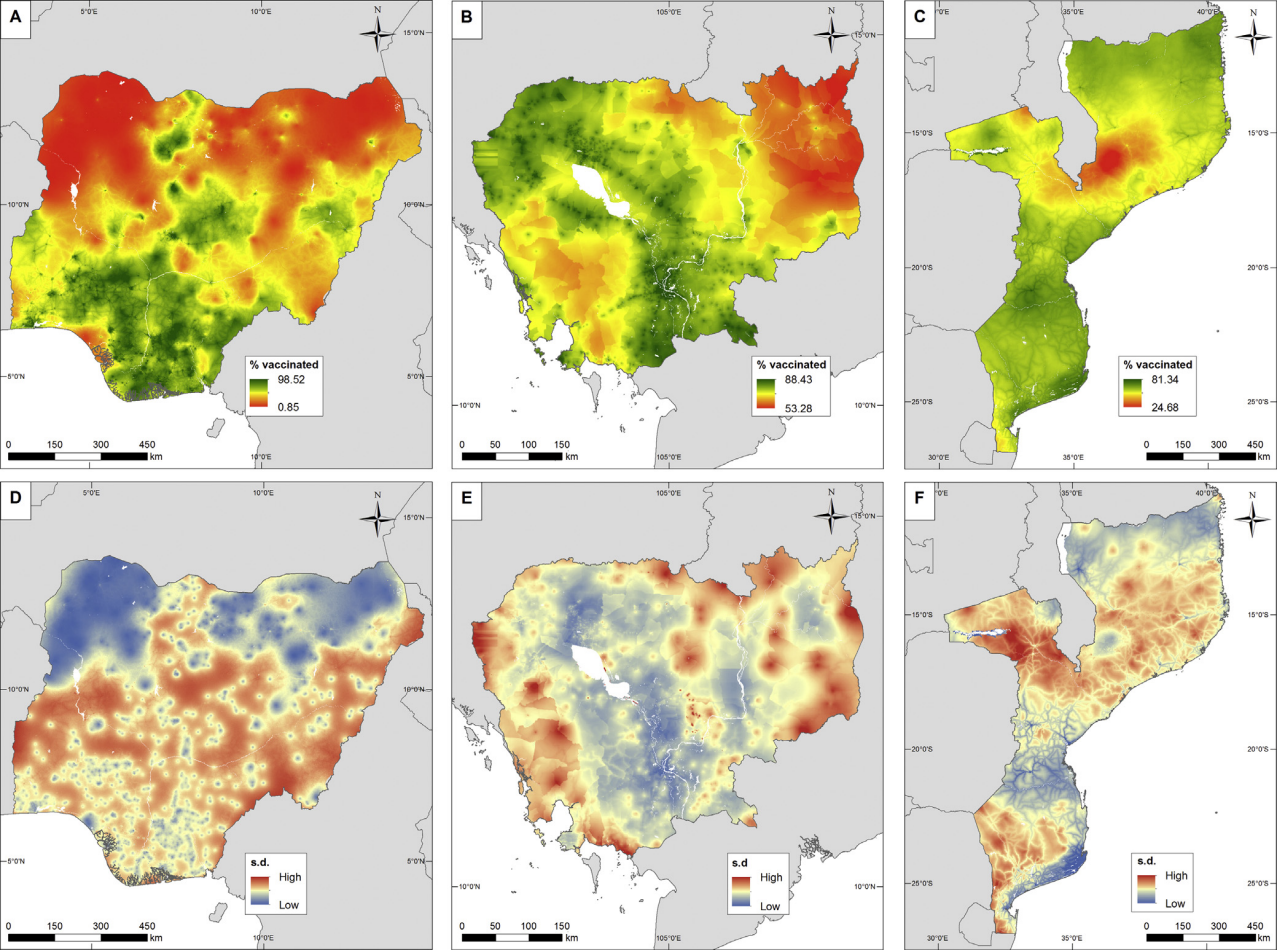
Predicted measles vaccination coverage in children under 5 years old at 1 × 1 km for (A) Nigeria 2013, (B) Cambodia 2014 and (C) Mozambique 2011, with associated uncertainty maps, measured as standard deviations, in (D), (E) and (F).

The outputs of the geostatistical modelling of measles vaccination coverage in children under 5 years of age are shown for the test countries in Fig. 2. The 1 × 1 km prediction maps in the top row highlight substantial geographic inequities in each country, though the differing colour scales should be accounted for when comparing between countries. The uncertainty maps in the bottom row show the standard deviation around per-grid square predictions. Where it is high, confidence in predictions is lower than where the standard deviation is low. Fig. 2A shows that in 2013 a substantial area of the north of Nigeria was predicted with high confidence (Fig. 2D) to have a negligibly low proportion of children under 5 years old vaccinated against measles, with spots of higher coverage only in the major towns and cities. This contrasts with the south of the country, where percentages vaccinated are significantly higher, though spatial heterogeneity and levels of uncertainty in predictions are generally higher. Fig. 2B And C highlight more favourable overall vaccination rates in Cambodia and Mozambique, but with clear regions of lower coverage. The percentages of under 5 year olds vaccinated drops to around 50–60% in northeast Cambodia and to 20–30% in north-central Mozambique.

**Fig. 3:**
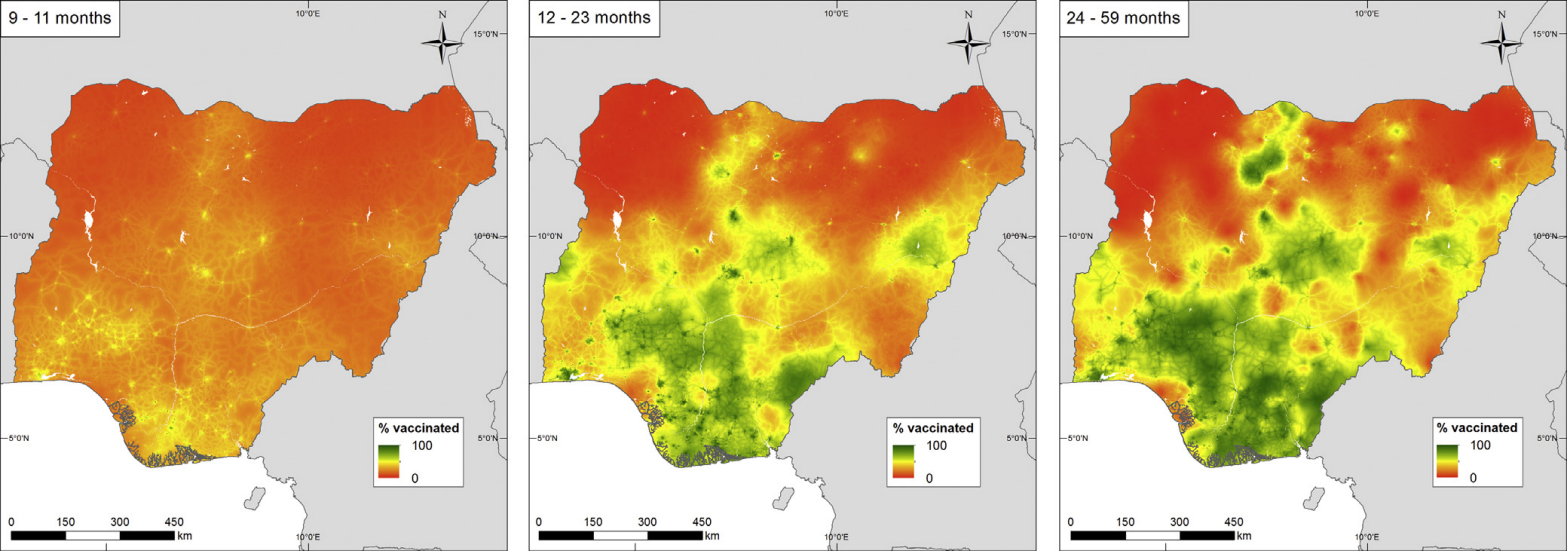
Predicted measles vaccination coverage at 1 × 1 km for Nigeria children (left) 9–11 months old, (middle) 12–23 months old, and (right) 24–59 months old.

An example output of the age-structured mapping for Nigeria in 2013 is shown in Fig. 3 (the same outputs for Cambodia and Mozambique are in supplemental material). Here, the spatial inequities in vaccination efforts are clear with the progression from 9–11 to 12–23, then 24–59 months showing rising proportions of the target population vaccinated against measles increasing in many areas of the country, particularly the south and central regions, but much of the northern areas remaining at zero or very low coverage rates.

### 3.4 Comparisons against existing national and regional estimates

**Fig. 4:**
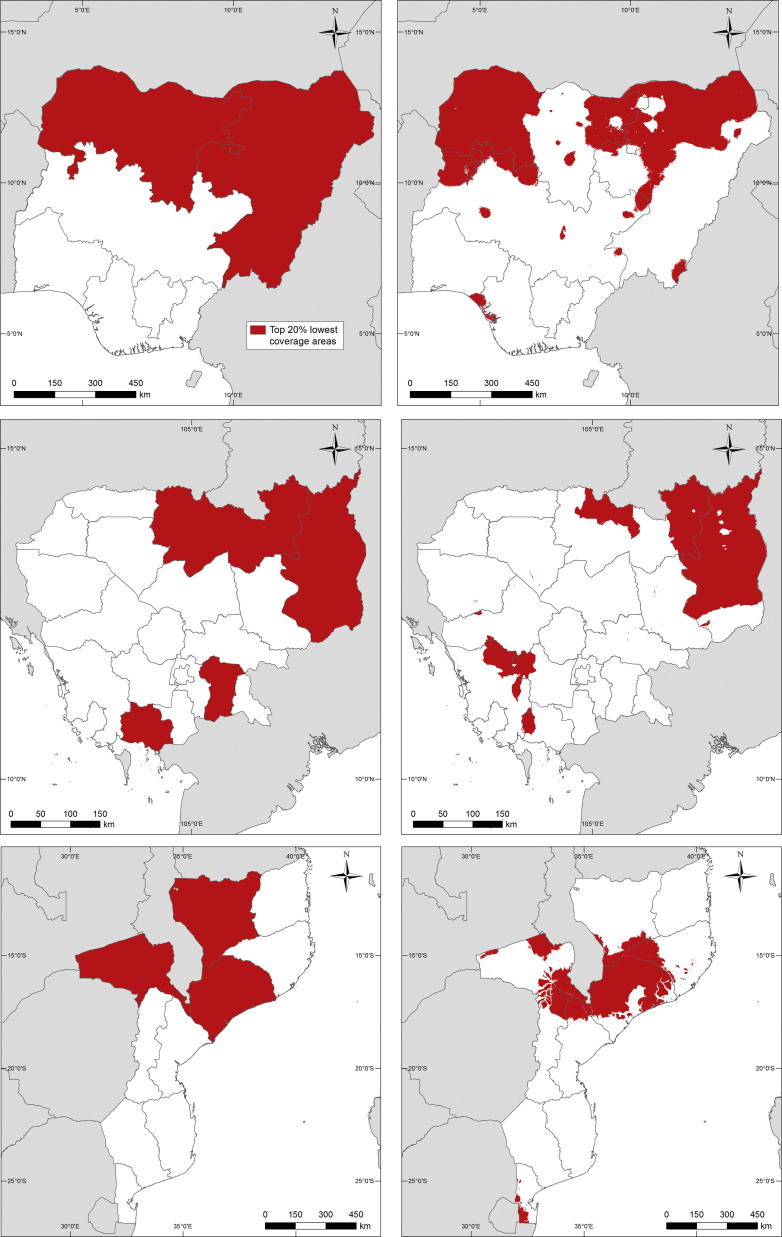
Maps showing those areas estimated to be the 20% lowest measles vaccination coverage areas in each country through using DHS region estimates (left column) and 1 × 1 km map estimates (right column).

Subnational assessments of vaccination coverage have typically been made using surveys such as the DHS aggregated to the provincial level. Using these estimates to prioritise vaccination efforts can mean that smaller coldspots of low coverage can be missed. Fig. 4 illustrates this through mapping the 20% of areas with the lowest estimated coverage through using DHS region estimates compared to the 1 × 1 km estimates. While similarities between the red areas are clear, significant differences are apparent as we move from large area summaries to finer scale mapping. In Nigeria in 2013, it is clear that the northwest and northeast areas have the lowest rates of coverage, but accounting for finer-scale heterogeneities reveal that the central-north and eastern regions are not consistently featured as part of the lowest 20% coverage areas, and that other ‘coldspots’ [2] appear that are masked through averaging across large areas. A similar story is evident for Cambodia and Mozambique, where there is general agreement between the regional and high resolution maps in terms of approximate low coverage areas, but the high resolution maps capture heterogeneities that are not apparent through aggregate summaries.

**Fig. 5:**
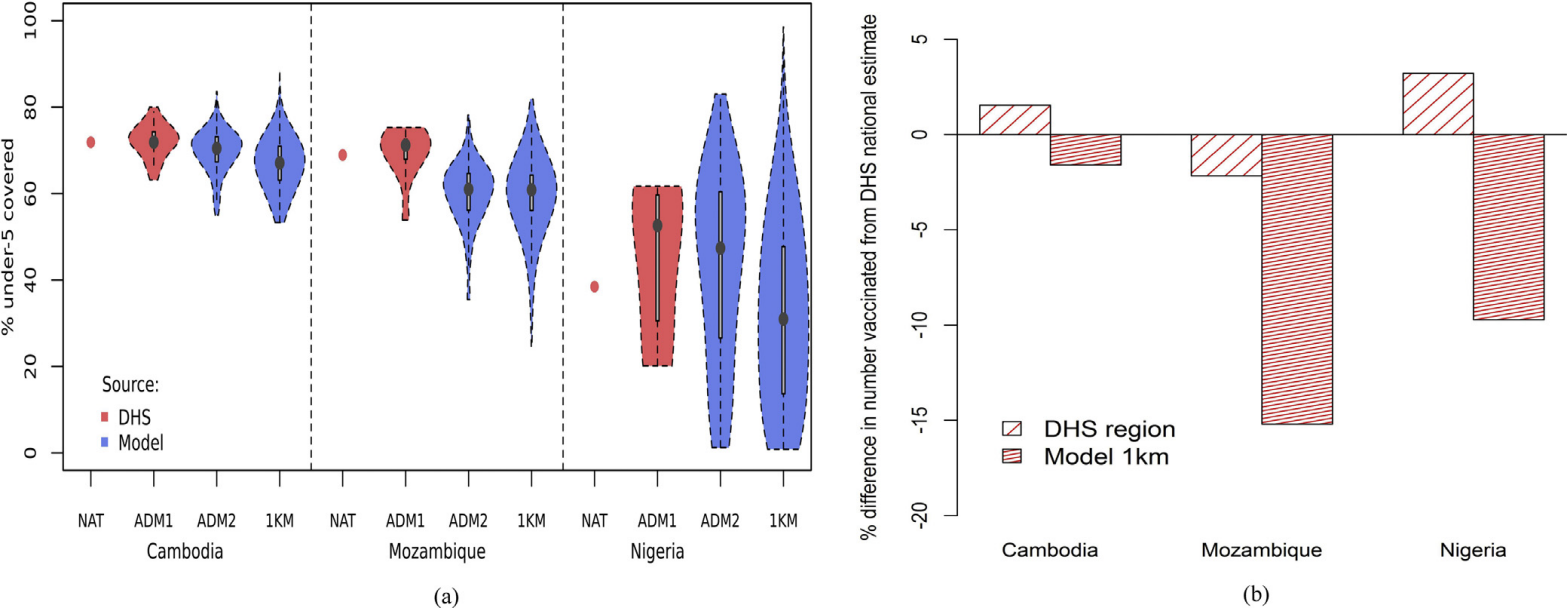
Differences in proportions and numbers of under 5 year old children vaccinated against measles through estimates constructed at varying levels of spatial aggregation. The variability in percentage covered estimates through national, sub-national administrative units and 1 × 1 km grid squares are shown in (a). The change in numbers vaccinated through moving from national to DHS region level and to 1 × 1 km grid squares is shown in (b). Further details are provided in supplementary materials (Tables S6–7).

At fine spatial scales, vaccination coverage is typically heterogeneous. This is often not captured by summaries of data at national or administrative unit level 1, and Fig. 5a illustrates this. Mapping vaccination coverage at high spatial resolution captures substantially more of the variability that exists across a country, with the figure comparing estimates made through traditional summaries of survey data at national and provincial levels (ADM1) in red, and estimates made at district (ADM2) and 1 × 1 km through the models outlined here shown in blue. The large area summaries do not capture the ‘coldspots’ of low coverage, and the greater variability around the mean is apparent at finer levels of spatial disaggregation. Moreover, the mean values and distributions around them are different as a result of the large area estimates summarising across units that cover urban and rural populations, with their typically higher and lower coverage rates, respectively. By capturing these heterogeneities with finer-scale mapping, the mean coverage values across all units typically become lower through larger numbers of rural units (with relatively low coverage) than urban (with higher coverage). These differences are also reflected in differences in estimates of the numbers of children under 5 years old vaccinated through moving from national to provincial to high resolution mapping (using gridded population datasets [10]), as shown in Fig. 5b. These differences are as large as 15% fewer children vaccinated for Mozambique compared to national level calculations. Maps of numbers of under 5 children unvaccinated are provided in supplemental materials.

**Fig. 6:**
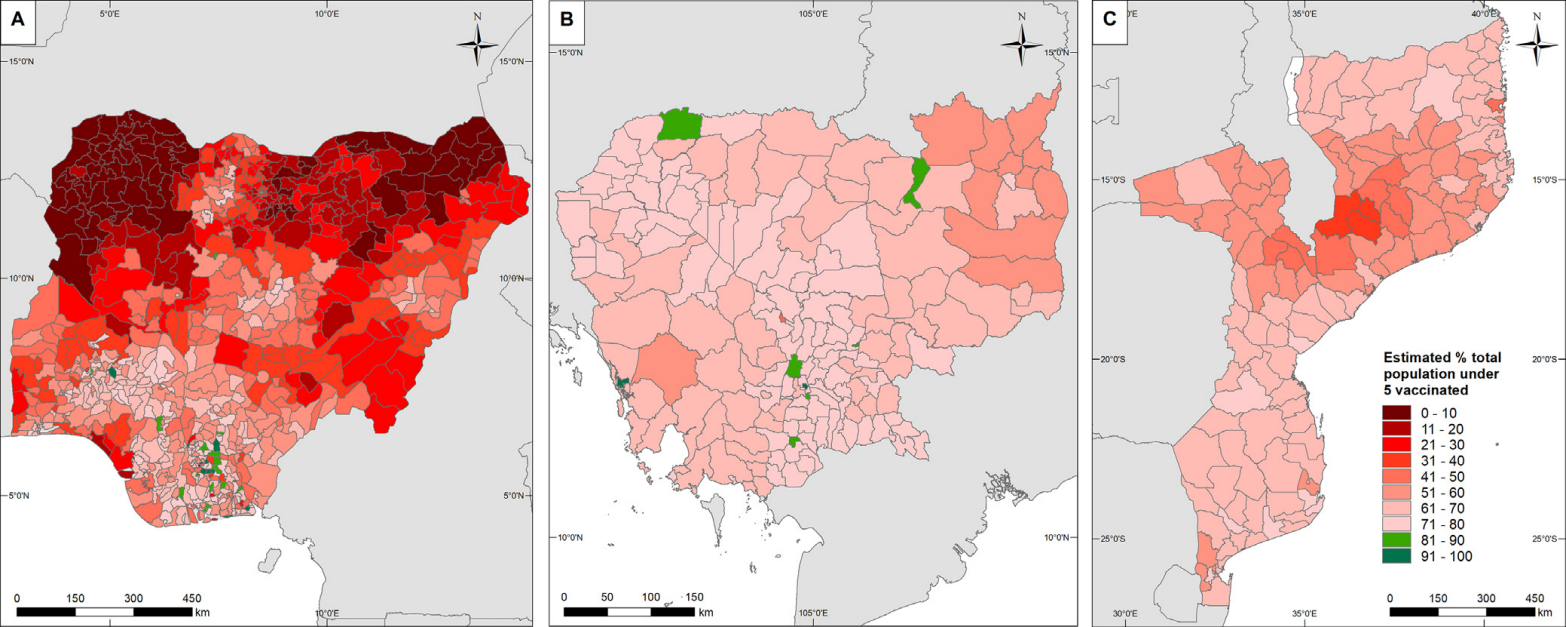
Maps of proportions of under 5 children estimated to be vaccinated against measles, with districts highlighted in green if they were above the WHO Global Vaccination Action Plan (GVAP) threshold of 80%, for (A) Nigeria in 2013, (B) Cambodia in 2014 and (C) Mozambique in 2011. (For interpretation of the references to colour in this figure legend, the reader is referred to the web version of this article.)

The Global Vaccine Action Plan (GVAP) sets out a target of reaching 80% coverage with all vaccines in all districts by 2020 [8]. The geostatistical mapping undertaken here provides a mechanism for measuring progress towards these targets through estimating coverage rates at fine spatial scales to enable district-level summaries to be produced, and combining them with gridded population data [10]. Although the GVAP targets relate to individual vaccines, the nature of the input survey data means that our assessment is based on the coverage of vaccination with at least the first dose of measles vaccine (MCV-1). Supplemental material shows examples of aggregation to different administrative levels and calculations of numbers unvaccinated (Figs. S5–8). Fig. 6 shows district-level estimates of the proportions of under 5 children estimated to be vaccinated against measles at the year of input survey data, and highlights that substantial efforts are needed in most places to meet the GVAP targets relating to measles vaccine. In Nigeria in 2013, Cambodia in 2014 and Mozambique in 2011, only 4%, 5% and 0% of districts respectively had coverage predicted to be >80%. While these numbers are low, it is clear however that the majority of districts in Cambodia and Mozambique were close to the 80% target, with coverage rates >60%. This is not the case though for Nigeria, where only relatively small regions in the south and centre either reached the 80% threshold or had coverage rates >60%.

## 4 Discussion

The launch of the Sustainable Development Goals with their ‘leave no one behind’ agenda [31], [32], the rise of disease eradication campaigns [1], [33], [34], [35], [36], [37], and tightening health budgets in many places [38], have contributed to the rise in thinking subnationally to improve measurements and target interventions more efficiently. This has driven an emphasis on geographically-located data collection and the development of methods to exploit this in the production of small area estimates in the health and development arena. Here we have shown the potential of such data and approaches in the context of childhood vaccination in uncovering heterogeneities previously masked by large area summaries, improving estimates of numbers covered and providing a framework for monitoring progress towards targets.

The modelling framework outlined here brings together freely-available datasets and open-source tools to produce 1 × 1 km estimates of vaccination coverage for key age groups, together with measures of uncertainty for these estimates. The validation statistics and relatively narrow uncertainty ranges show the strong predictive power of the models, and highlight how variabilities in coverage rates can be captured at local scales using just 4–5 geospatial covariates. While some consistencies in covariate selection were seen (e.g. remoteness), different sets of geospatial datasets were selected for the best model for each of the three test countries. This suggests potentially differing drivers of vaccination coverage distributions, and that challenges may arise in building more universal multi-country or global models, as highlighted with other health and development metrics [9].

It is clear from the analyses here that high levels of vaccination coverage measured at national and regional levels still mask significant spatial heterogeneities that represent a risk for outbreaks. While the focus has been on measles vaccination as a test case, many of the methods and findings translate to other vaccine preventable diseases. Population movements linking up areas of low coverage and high population densities could lead to the persistence of transmission even with comprehensive vaccination campaigns in other areas. An illustration of the potential for improved prioritization of target areas through, for example, enhancing routine health care services or spatially-focused SIA campaigns is shown in Fig. 4, where refined spatial detail aids in identification of coldspots that are missed at regional scales. Moreover, the value of more spatially precise estimates in improving the precision of mapping and estimation of susceptible numbers is illustrated through the differences seen in Fig. 5 through switching from large area to 1 × 1 km estimates.

While the modelling framework and results presented here show strong potential, it is clear that limitations do exist. The model validation statistics show that coverage rates were not predicted perfectly, particularly where sample sizes were small. The occurrence of smaller sample sizes at some cluster locations results from a combination of factors, such as the survey design (DHS survey samples are selected to ensure representativeness at coarser administrative areas than the cluster level) and disproportionate distributions of children under 5 years within the clusters. An examination of the data sets used in model fitting revealed no marked pattern, such as urban-rural differences, in sample size distribution in all three countries. Statistically, the estimation of binomial probabilities with small numbers of trials leads to poorer predictive power and greater uncertainty (see, e.g. [39]), as is the case in some of the models fitted here. However, these inaccuracies are aptly captured by the uncertainty (standard deviation) maps (Fig. 2) and the validation statistics (Table S5) reported and can form a basis for guiding additional targeted data collection. The covariate layers used cover a wide range of factors associated with vaccination coverage, but data on many others, such as access to healthcare, education, literacy, health facility staffing levels, and vaccine stocks were not available to improve outputs further. Additionally, the approaches outlined here produce maps that are necessarily tied to the date of the coverage surveys. Obtaining more recent maps requires either more recent survey data, or the implementation of demographic and epidemiological modelling techniques, which are the focus of ongoing work [2], [40].

The methods presented here provide a robust approach for mapping vaccination coverage rates at high spatial resolution, and there are various avenues for future improvements and new directions. Here measles vaccination in three countries was used to test and demonstrate approaches, but the potential exists to examine the applicability of these approaches to a range of other childhood vaccinations, and expand to new settings. With national household surveys forming a snapshot in countries undergoing rapid demographic and health changes, obtaining contemporary estimates of vaccination coverage and numbers susceptible to disease will require the integration of additional forms of data, such as subnational fertility rates and the timings and locations of supplemental immunization activities into demographic and disease transmission models. This work forms part of a larger effort to undertake this, resulting in high resolution estimates of susceptibility to guide strategies [2], [40]. Additional forms of data can also add value in strategic planning on mechanisms of delivery to reach those areas and populations with the lowest rates of vaccination coverage. These include geospatial treatment seeking and health facility catchment models [41], [42] and mobile network data to quantify seasonally varying vaccine demands at health facilities [43], and identify and map mobile populations [44].
