# Supplementary material for: High resolution age-structured mapping of childhood vaccination coverage in low and middle income countries
Source: Vaccine. 2018 Mar 14;36(12):1583–91. doi: 10.1016/j.vaccine.2018.02.020 (PMC6344781; doi:10.1016/j.vaccine.2018.02.020)
Supplement: Supplementary data 1 [file mmc1.docx]

**Supplemental materials for “High resolution age-structured mapping of childhood vaccination coverage in low and middle income countries”**

C. Edson Utazi ^a,b^, Julia Thorley ^a^, Victor A. Alegana ^a,c^, Matthew J. Ferrari ^d^, Saki Takahashi ^e^, C. Jessica E. Metcalf ^e^, Justin Lessler ^f^, and Andrew J. Tatem ^a,c^

^a^WorldPop, Department of Geography and Environment, University of Southampton, Southampton, SO17 1BJ, UK

^b^Southampton Statistical Sciences Research Institute, University of Southampton, Southampton, SO17 1BJ, UK

^c^Flowminder Foundation, Stockholm SE-11355, Sweden

^d^Center for Infectious Disease Dynamics, The Pennsylvania State University, State College, Pennsylvania 16802, USA

^e^Department of Ecology and Evolutionary Biology, Princeton University, Princeton, New Jersey 08544, USA

^f^Department of Epidemiology, Johns Hopkins Bloomberg School of Public Health, Baltimore, Maryland 21205, USA

**Introduction**

These supplemental materials accompany the main paper. Here, we provide further details of the methods section and additional figures and tables.

**Covariate data processing and selection**

In most cases, the most recent versions of the geospatial covariates listed in Table S1 were used in the analysis. Wherever possible, the versions of the datasets closest to the year of the national DHS survey were used. The format (raster, vector) and spatial resolution varied across the original assembled covariate datasets, and therefore these were standardized to a common format. Using ArcGIS 10.4, we constructed a standardised set of gridded covariate layers at a 1x1 km resolution for all the countries. To extract the covariate data at the locations of the DHS clusters from the standardized layers, 5 km buffers were created around clusters in rural areas, and 2 km buffers around clusters in urban areas, to account for the displacement of the clusters, as specified by the DHS (1). For continuous covariates, the minimum, maximum, mean and where possible, median values for the grid cells falling within the buffer were extracted, whereas for categorical covariates (e.g. land cover), the majority class was extracted. Where datasets were binary, the maximum value present was extracted in order to identify whether any pixels corresponding to the covariate were present at the cluster location. However, for the sake of uniformity, only the means of the continuous covariates were retained in the analysis.

As is standard practice in spatial modelling, covariate selection was performed in a non-spatial, frequentist framework using binomial generalized linear models (GLMs) to determine the best combination of covariates for modelling vaccination coverage. We first implemented an age-specific covariate selection procedure for each country before choosing a uniform set of covariates for the country.

For a given age group, the relationships between each covariate and the logit of the observed vaccination probabilities were first examined for linearity using scatter plot diagrams. The log transformation was applied to improve the relationships and the distributions of the covariates where evidence of non-linearity and significant skewness occurred. To guard against the problem of multicollinearity, the correlations between the covariates were calculated and we chose between highly correlated covariates ($\rho\geq0.80$) using the AIC (Akaike Information Criterion) statistics of the corresponding single covariate binomial regression models. The variance inflation factors (VIF) (2) of the covariates were also calculated as an additional step to detect collinear variables. Again, the most important covariates within sets of correlated covariates with high VIF values (> 4.0) were selected using the AIC criterion. Finally, the p-value approach (relative to backward elimination (3)) coupled with expert judgement was used to determine the relevant set of covariates for modelling vaccination probability for the given age group. Multiple approaches to covariate selection exist, and here this method was used because of the large set of potential covariates and the need to identify those that were informative and statistically significant to ease the following covariate selection step. Once the relevant set of age-specific covariates was identified, these were then considered for inclusion in the uniform set of covariates for all age groups for a given country. In determining this uniform set of covariates, preference was given to the covariates that were selected across at least two age groups in most cases. The use of a uniform set of covariates in the analysis facilitates comparisons across the age groups.

**Model fitting and validation details**

To complete the Bayesian specification of the geostatistical model given in equation (1) in the main paper we placed weakly informative priors on the parameters. These were: a Normal (0,10^5^) prior on the regression coefficients ($\boldsymbol{\beta}$), an Inverse Gamma (2, 1) on $\sigma^{2}$ and a Uniform (0.02, 70) on $\phi$ - defining an effective spatial range (i.e. distance at which spatial dependence is negligible, calculated as $-log(0.05)/\phi$ in decimal degrees) between approximately 5 km and 17,000 km. For each analysis, the MCMC algorithm was run for 100,000 iterations, with the first 10,000 discarded as burn-in. The chains were examined for convergence by visual inspection and using other convergence diagnostics. Aggregation of the predicted vaccination probabilities to various administrative levels was carried out by Monte Carlo integration using the 1x1 km prediction grids (see (4), Chapter 7).The model was implemented using the spBayes package in R (5, 6).

For model validation, we employed the hold-out method of cross-validation, setting aside a 10% subset of the data each time. Using the hold-out data from $m$ cluster locations, we computed the following model evaluation criteria: percentage bias (% Bias=$100\times\sum_{i} \left( \hat{p}_{i} -p_{i} \right)/\sum_{i} p_{i}$), validation mean square error (VMSE= $\sum_{i} \left( \hat{p}_{i} -p_{i} \right)^{2}/m$ ) and the nominal coverage of the 95% prediction intervals $(100\times\sum_{i} {I\left( l_{i}\leq\hat{y}_{i}\leq u_{i} \right)}/m)$; where $\hat{p}_{i}$ denotes the predicted probability for the *i*th hold-out location, $l_{i} \mathrm{and} u_{i}$are the lower and upper limits of the prediction interval and $I(.)$ is an indicator function. The posterior estimates of the numbers of children vaccinated ($\hat{y}_{i};i=1,\ldots,m$) were used to calculate the nominal coverage due to the erratic behaviour caused by proximity to the endpoints (i.e. 0 and 1) often encountered when using binomial probabilities (see, e.g., (7)). In the calculations, the estimated lower (2.5th percentile) and upper (97.5th percentile) credible intervals were set equal to the observed values where the latter occurred at the endpoints. The closer the nominal coverage is to its true value (i.e. 95%), the better the model. Smaller values of % Bias (in absolute value) and VMSE indicate a better model performance. Lastly, the coefficients of determination (R^2^) of the fitted models were calculated as the square of the Pearson’s correlation between the observed and predicted probabilities.

**Figures and tables**

**
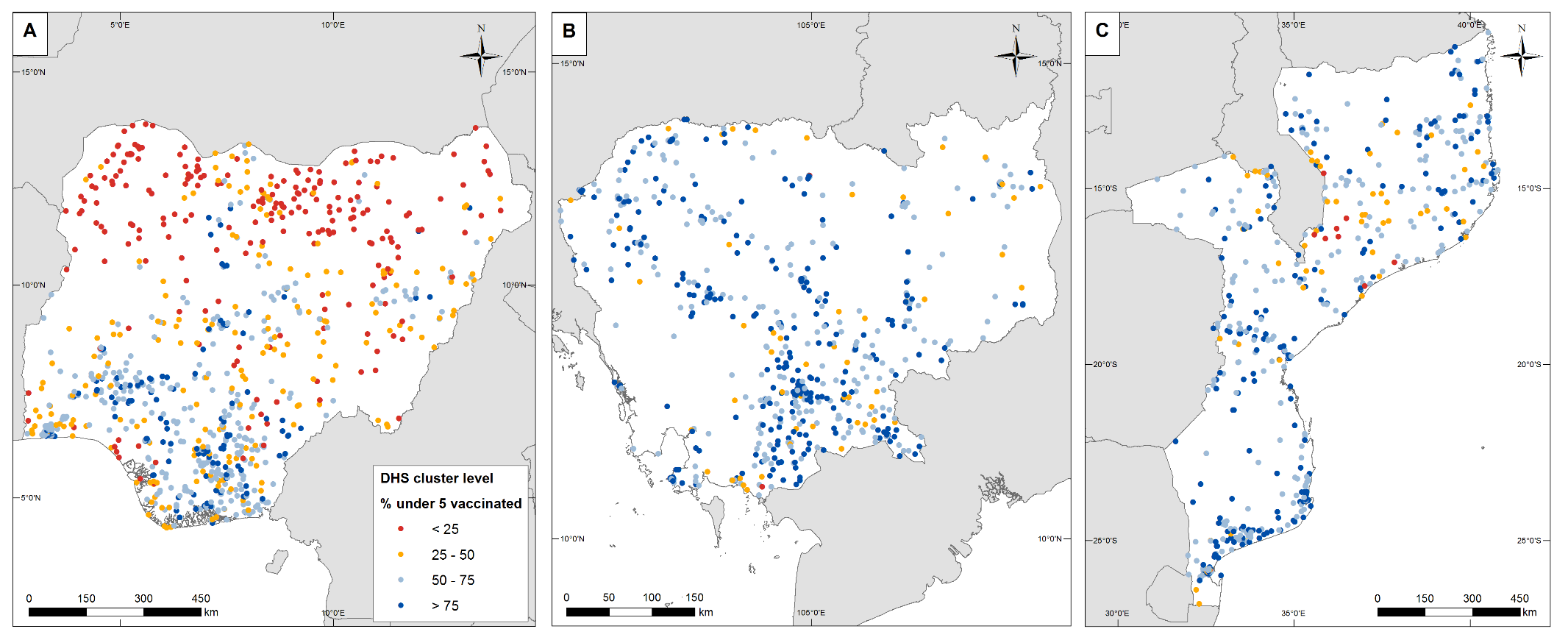
**

**Figure S1.** Spatial distribution of measles vaccination coverage for children under 5 years old as recorded at the DHS cluster level for (a) Nigeria 2013, (b) Cambodia 2014 and (c) Mozambique 2011. The colour of the dots indicates the percentage of children in each cluster estimated to be vaccinated according to the survey data.

**Table S1: Geospatial covariates assembled for testing within the modelling framework**

| **Category** | **Covariates** | **Sources** |
| --- | --- | --- |
| Demographic | Population density, Population counts, Ethnicity, Family groups | WorldPop Project ([www.worldpop.org](http://www.worldpop.org/)), GPW v4 and Global Rural-Urban Mapping Project (GRUMP) v.1. SEDAC/CIESEN (<http://sedac.ciesin.columbia.edu>), ETH Zurich (<https://icr.ethz.ch/data/greg/>), People's Atlas of Africa (<http://worldmap.harvard.edu/data/geonode:Ethnicity_Africa_2001_Felix_Scan>) |
| Remoteness | Travel time to major cities, Distances to urban areas, Railways, Roads, Infrastructure, Waterbodies, Waterways, Highways, Places (towns, cities and villages), Residential areas, Protected areas, Conflicts, Conflict locations | European Commission Joint Research Centre (<http://forobs.jrc.ec.europa.eu/products/gam>), input data from Open Street map ([www.openstreetmap.org](http://www.openstreetmap.org/)), WDPA (<http://protectedplanet.net/>) ACLED (<http://www.acleddata.com/data/>) |
| Climate and Environment | Temperature, Precipitation, Aridity, Evapotranspiration, MODIS net primary production, MODIS Enhanced Vegetation Index (EVI), Mid-infrared index, MODIS Land Surface Temperature (LST) | WorldClim ([www.worldclim.org](http://www.worldclim.org/)), Climate Hazards Group InfraRed Precipitation with Station data (CHIRPS) (<http://chg.geog.ucsb.edu/data/chirps/>), Consortium for Spatial Information (CGIAR-CSI) ([www.cgiar-csi.org](http://www.cgiar-csi.org/)), MODIS - NASA (https://modis.gsfc.nasa.gov/data/) |
| Topography | Elevation, Slope | US Geological Survey (USGS) (<http://eros.usgs.gov/elevation-products>) |
| Land use/cover | Protected areas, Urban areas, Settlements, Night-time lights, Land cover | WDPA (<http://protectedplanet.net/>), DLR German Aerospace Centre (<http://www.dlr.de/>), SAGE / Schneider et al. 2010 (<https://nelson.wisc.edu/sage/data-and-models/schneider.php>), NOAA VIIRS ([ngdc.noaa.gov/eog](http://ngdc.noaa.gov/eog)), ESA Globcover (<http://due.esrin.esa.int/page_globcover.php>), National Geomatics Center of China Globeland 30 ([www.globallandcover.com/](http://www.globallandcover.com/)), EU JRC GLC2000 (<http://forobs.jrc.ec.europa.eu/products/glc2000/glc2000.php>) |
| Economic | Poverty, Economic index | Tatem AJ, Gething PW, Bhatt S, Weiss D and Pezzulo C (2013) Pilot high resolution poverty maps, University of Southampton/Oxford.  (<http://www.worldpop.org.uk/resources/docs/WorldPop-poverty-mapping-methods.pdf>)  G-Econ Project, Yale University, 2009. (<http://gecon.yale.edu/>) |
| Agriculture | Poultry, Cattle, Pigs, Sheep, Goats, Land suitability for pasture and rainfed crops | FAO in collaboration with the Environmental Research Group Oxford (ERGO) ([http://livestock.geo-wiki.org](http://livestock.geo-wiki.org/)), FAO FGGD (<http://geonetwork3.fao.org/fggd/>) |

**Table S2. Estimates of the parameters of the fitted models for Cambodia. Reported are the posterior means, standard deviations (SD) and quantiles (2.5%, 50% and 97.5%) of the regression coefficients and the parameters of the spatial random effect,** $\boldsymbol{w}$**.**

| **Age 0-8 months** | | | | | |
| --- | --- | --- | --- | --- | --- |
| **Parameter** | **Mean** | **SD** | **2.5%** | **50%** | **97.5%** |
| (Intercept) | -11.8064 | 4.3140 | -20.2324 | -11.9452 | -3.1779 |
| log (travel time) | 0.3928 | 0.3317 | -0.2590 | 0.3978 | 1.0750 |
| log (population density) | 0.2823 | 0.2211 | -0.1590 | 0.2817 | 0.7219 |
| log (distance to residential areas) | -0.1210 | 0.1819 | -0.4600 | -0.1244 | 0.2557 |
| log (distance to infrastructures) | 0.6710 | 0.3100 | 0.0981 | 0.6572 | 1.3963 |
| Partial sill ($\sigma^{2}$) | 0.5571 | 0.3523 | 0.1611 | 0.4683 | 1.5297 |
| Spatial decay ($\phi$)* | 38.7897 | 18.4762 | 7.4649 | 39.3848 | 68.5895 |
| **Age 9-11 months** | | | | | |
| **Parameter** | **Mean** | **SD** | **2.5%** | **50%** | **97.5%** |
| (Intercept) | 9.5766 | 2.1114 | 5.8913 | 9.3586 | 13.7567 |
| log (travel time) | -0.2212 | 0.1643 | -0.5463 | -0.2158 | 0.1016 |
| log (population density) | -0.2153 | 0.1196 | -0.4655 | -0.2105 | 0.0076 |
| log (distance to residential areas) | -0.3950 | 0.1315 | -0.6607 | -0.3907 | -0.1463 |
| log (distance to infrastructures) | -0.3649 | 0.1601 | -0.7012 | -0.3621 | -0.0590 |
| Partial sill ($\sigma^{2}$) | 0.3842 | 0.1974 | 0.1489 | 0.3369 | 0.8747 |
| Spatial decay ($\phi$)* | 41.1202 | 17.5276 | 9.5588 | 41.8836 | 68.6109 |
| **Age 12-23 months** | | | | | |
| **Parameter** | **Mean** | **SD** | **2.5%** | **50%** | **97.5%** |
| (Intercept) | 2.5041 | 1.9339 | -1.6423 | 2.6123 | 5.9893 |
| log (travel time) | 0.0036 | 0.1415 | -0.2596 | 0.0035 | 0.2864 |
| log (population density) | 0.2065 | 0.1034 | 0.0199 | 0.2008 | 0.4071 |
| log (distance to residential areas) | -0.0472 | 0.1006 | -0.2407 | -0.0501 | 0.1556 |
| log (distance to infrastructures) | -0.1663 | 0.1246 | -0.3962 | -0.1638 | 0.0706 |
| Partial sill ($\sigma^{2}$) | 0.7977 | 0.2886 | 0.3280 | 0.7725 | 1.4154 |
| Spatial decay ($\phi$)* | 25.1495 | 20.5905 | 2.0853 | 18.5527 | 66.5725 |
| **Age 24-59 months** | | | | | |
| **Parameter** | **Mean** | **SD** | **2.5%** | **50%** | **97.5%** |
| (Intercept) | 2.3057 | 1.4386 | -0.4114 | 2.2957 | 5.3868 |
| log (travel time) | -0.2354 | 0.1109 | -0.4673 | -0.2313 | -0.0297 |
| log (population density) | 0.2170 | 0.0751 | 0.0711 | 0.2155 | 0.3655 |
| log (distance to residential areas) | -0.0156 | 0.0896 | -0.2297 | -0.0081 | 0.1375 |
| log (distance to infrastructures) | 0.0476 | 0.0878 | -0.1218 | 0.0464 | 0.2165 |
| Partial sill ($\sigma^{2}$) | 0.8623 | 0.1924 | 0.5242 | 0.8477 | 1.2825 |
| Spatial decay ($\phi$)* | 44.8869 | 15.5358 | 16.6501 | 45.6679 | 68.9980 |

*The estimated decay parameters correspond to effective spatial ranges of 8 km, 8 km, 18 km and 7 km, respectively.

**Table S3. Estimates of the parameters of the fitted models for Mozambique. Reported are the posterior means, standard deviations (SD) and quantiles (2.5%, 50% and 97.5%) of the regression coefficients and the parameters of the spatial random effect,** $\boldsymbol{w}$**.**

| **Age 0-8 months** | | | | | |
| --- | --- | --- | --- | --- | --- |
| **Parameter** | **Mean** | **SD** | **2.5%** | **50%** | **97.5%** |
| (Intercept) | -1.9014 | 2.9201 | -7.5113 | -1.8051 | 4.1899 |
| log (travel time) | -0.1477 | 0.1049 | -0.3497 | -0.1489 | 0.0574 |
| Precipitation | 0.0061 | 0.0078 | -0.0095 | 0.0061 | 0.0210 |
| Evapotranspiration | -0.0005 | 0.0016 | -0.0034 | -0.0005 | 0.0025 |
| log (MODIS net primary production) | -0.7717 | 0.7269 | -2.2346 | -0.7592 | 0.6002 |
| Partial sill ($\sigma^{2}$) | 1.2464 | 0.4996 | 0.5536 | 1.1488 | 2.4334 |
| Spatial decay ($\phi$)* | 24.1822 | 21.2486 | 1.5957 | 16.7587 | 67.5523 |
| **Age 9-11 months** | | | | | |
| **Parameter** | **Mean** | **SD** | **2.5%** | **50%** | **97.5%** |
| (Intercept) | 3.4640 | 1.7698 | 0.0572 | 3.3636 | 7.1493 |
| log (travel time) | -0.2338 | 0.0918 | -0.4143 | -0.2351 | -0.0561 |
| Precipitation | -0.0054 | 0.0054 | -0.0158 | -0.0056 | 0.0053 |
| Evapotranspiration | -0.0010 | 0.0010 | -0.0031 | -0.0009 | 0.0009 |
| log (MODIS net primary production) | -0.4367 | 0.5849 | -1.5810 | -0.4442 | 0.7101 |
| Partial sill ($\sigma^{2}$) | 0.4193 | 0.1939 | 0.1658 | 0.3780 | 0.9095 |
| Spatial decay ($\phi$)* | 31.6977 | 18.9877 | 4.9874 | 28.5481 | 67.5935 |
| **Age 12-23 months** | | | | | |
| **Parameter** | **Mean** | **SD** | **2.5%** | **50%** | **97.5%** |
| (Intercept) | 5.6503 | 1.8226 | 3.0931 | 5.3180 | 10.1999 |
| log (travel time) | -0.2809 | 0.0775 | -0.4321 | -0.2823 | -0.1276 |
| Precipitation | -0.0060 | 0.0049 | -0.0156 | -0.0061 | 0.0041 |
| Evapotranspiration | -0.0013 | 0.0010 | -0.0037 | -0.0011 | 0.0002 |
| log (MODIS net primary production) | 0.2953 | 0.5570 | -0.8601 | 0.3228 | 1.3535 |
| Partial sill ($\sigma^{2}$) | 1.0824 | 0.2473 | 0.6682 | 1.0567 | 1.6278 |
| Spatial decay ($\phi$)* | 22.9603 | 14.7969 | 6.2433 | 18.0310 | 62.3810 |
| **Age 24-59 months** | | | | | |
| **Parameter** | **Mean** | **SD** | **2.5%** | **50%** | **97.5%** |
| (Intercept) | 5.2505 | 1.3627 | 2.9568 | 5.1501 | 8.1770 |
| log (travel time) | -0.0392 | 0.0773 | -0.1949 | -0.0376 | 0.1037 |
| Precipitation | -0.0241 | 0.0059 | -0.0369 | -0.0238 | -0.0132 |
| Evapotranspiration | -0.0007 | 0.0007 | -0.0020 | -0.0007 | 0.0005 |
| log (MODIS net primary production) | 0.8597 | 0.3859 | 0.0824 | 0.8658 | 1.6026 |
| Partial sill ($\sigma^{2}$) | 1.4181 | 0.2089 | 1.0576 | 1.4030 | 1.8716 |
| Spatial decay ($\phi$)* | 5.2886 | 2.6868 | 2.6274 | 4.7204 | 12.1269 |

*The estimated decay parameters correspond to effective spatial ranges of 14 km, 12 km, 19 km and 71 km, respectively.

**Table S4. Estimates of the parameters of the fitted models for Nigeria. Reported are the posterior means, standard deviations (SD) and quantiles (2.5%, 50% and 97.5%) of the regression coefficients and the parameters of the spatial random effect,** $\boldsymbol{w}$**.**

| **Age 0-8 months** | | | | | |
| --- | --- | --- | --- | --- | --- |
| **Parameter** | **Mean** | **SD** | **2.5%** | **50%** | **97.5%** |
| (Intercept) | -4.4938 | 1.7158 | -7.6002 | -4.5092 | -1.3268 |
| Poverty | 1.3789 | 1.9611 | -2.2979 | 1.3619 | 4.9478 |
| Aridity | 0.0001 | 0.0000 | 0.0000 | 0.0001 | 0.0002 |
| log(Night-time lights) | 0.1209 | 0.2212 | -0.3028 | 0.1199 | 0.5528 |
| log(travel time) | -0.2427 | 0.1108 | -0.4536 | -0.2442 | -0.0243 |
| EVI | -0.0279 | 1.7522 | -3.4989 | -0.0362 | 3.3857 |
| Partial sill ($\sigma^{2}$) | 1.6819 | 0.4861 | 0.8122 | 1.6425 | 2.7413 |
| Spatial decay ($\phi$)* | 41.2561 | 16.3978 | 12.7703 | 41.0019 | 68.4622 |
| **Age 9-11 months** | | | | | |
| **Parameter** | **Mean** | **SD** | **2.5%** | **50%** | **97.5%** |
| (Intercept) | -0.6702 | 1.1697 | -2.7031 | -0.7398 | 1.6967 |
| Poverty | -1.8040 | 1.1912 | -4.1874 | -1.7904 | 0.3393 |
| Aridity | 1.97x10^-5^ | 2.73 x10^-5^ | -3.37 x10^-5^ | 1.97 x10^-5^ | 7.54 x10^-5^ |
| log(Night-time lights) | 0.3616 | 0.1849 | -0.0110 | 0.3666 | 0.7071 |
| log(travel time) | -0.1650 | 0.1005 | -0.3753 | -0.1641 | 0.0219 |
| EVI | 4.4929 | 1.5569 | 1.4255 | 4.4870 | 7.5622 |
| Partial sill ($\sigma^{2}$) | 1.3990 | 0.3540 | 0.8020 | 1.3710 | 2.1976 |
| Spatial decay ($\phi$)* | 47.2393 | 15.7745 | 14.3480 | 49.8516 | 69.0363 |
| **Age 12-23 months** | | | | | |
| **Parameter** | **Mean** | **SD** | **2.5%** | **50%** | **97.5%** |
| (Intercept) | 1.7058 | 1.4841 | -1.1942 | 1.6435 | 4.7938 |
| Poverty | -4.8386 | 1.5090 | -8.2335 | -4.8413 | -1.9660 |
| Aridity | 7.62x10^-5^ | 5.65 x10^-5^ | -3.21 x10^-5^ | 7.51 x10^-5^ | 0.0002 |
| log(Night-time lights) | 0.7258 | 0.1309 | 0.4753 | 0.7234 | 0.9901 |
| log(travel time) | -0.0944 | 0.0730 | -0.2333 | -0.0968 | 0.0503 |
| EVI | 5.1894 | 1.2722 | 2.7704 | 5.1162 | 7.8071 |
| Partial sill ($\sigma^{2}$) | 2.1976 | 0.5597 | 1.4462 | 2.1102 | 3.5647 |
| Spatial decay ($\phi$)* | 1.1777 | 0.3170 | 0.5847 | 1.1675 | 1.8369 |
| **Age 24-59 months** | | | | | |
| **Parameter** | **Mean** | **SD** | **2.5%** | **50%** | **97.5%** |
| (Intercept) | 0.0686 | 0.7862 | -1.2901 | -0.1035 | 1.7370 |
| Poverty | -1.9365 | 0.9161 | -3.9308 | -1.7892 | -0.4501 |
| Aridity | 0.0001 | 3.65 x10^-5^ | 6.86 x10^-5^ | 0.0001 | 0.0002 |
| log(Night-time lights) | 0.3983 | 0.1065 | 0.1867 | 0.4007 | 0.5981 |
| log(travel time) | -0.2597 | 0.0599 | -0.3695 | -0.2596 | -0.1408 |
| EVI | 4.2467 | 1.3029 | 1.6006 | 4.2514 | 6.5761 |
| Partial sill ($\sigma^{2}$) | 2.3571 | 0.3128 | 1.8349 | 2.3273 | 3.0622 |
| Spatial decay ($\phi$)* | 2.3111 | 0.3925 | 1.6101 | 2.2894 | 3.1466 |

These correspond to effective spatial ranges of 8 km, 7 km, 286 km and 146 km, respectively.

**Table S5:** **Summary of validation statistics (95% nominal coverage, %bias and validation mean square error (VMSE)) based on 10% hold out data for the three test countries and the R^2^ values of the fitted models**

| **Country** | **Age (months)** | | | | |
| --- | --- | --- | --- | --- | --- |
|  | **0-8** | **9-11** | **12-23** | **24-59** | **0-59** |
|  | **Coverage (%)** | | | | |
| Nigeria | 95.63 | 94.12 | 92.78 | 94.89 | 93.75 |
| Cambodia | 98.20 | 98.54 | 94.37 | 92.11 | 91.95 |
| Mozambique | 97.47 | 95.63 | 94.02 | 92.11 | 91.13 |
|  | **% Bias** | | | | |
| Nigeria | -8.20 | 2.80 | -2.63 | -0.65 | -0.83 |
| Cambodia | -5.77 | -1.32 | -0.94 | -0.96 | -1.09 |
| Mozambique | -2.87 | -2.84 | -0.07 | -0.27 | -0.69 |
|  | **VMSE** | | | | |
| Nigeria | 0.01 | 0.17 | 0.07 | 0.02 | 0.03 |
| Cambodia | 0.00 | 0.23 | 0.11 | 0.02 | 0.03 |
| Mozambique | 0.01 | 0.14 | 0.04 | 0.02 | 0.02 |
|  | **R^2^** | | | | |
| Nigeria | 0.72 | 0.66 | 0.78 | 0.95 | 0.95 |
| Cambodia | 0.12 | 0.26 | 0.51 | 0.73 | 0.65 |
| Mozambique | 0.56 | 0.34 | 0.67 | 0.77 | 0.78 |


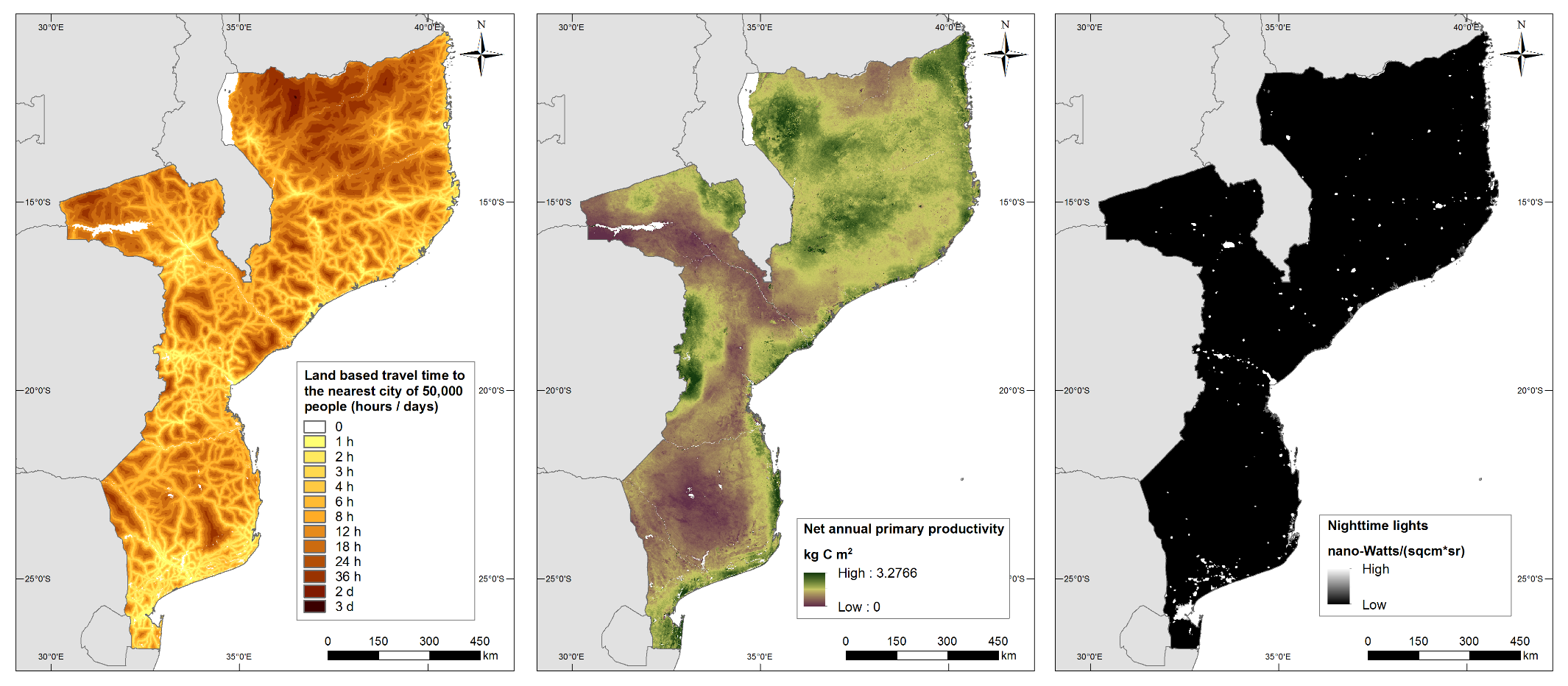


**Figure S2.** Examples of some of the covariates tested for inclusion in the geostatistical model. Left to right: Land based travel time to the nearest city of > 50,000 people; net annual primary productivity; night-time lights satellite imagery. These are for illustration and are shown for Mozambique only.


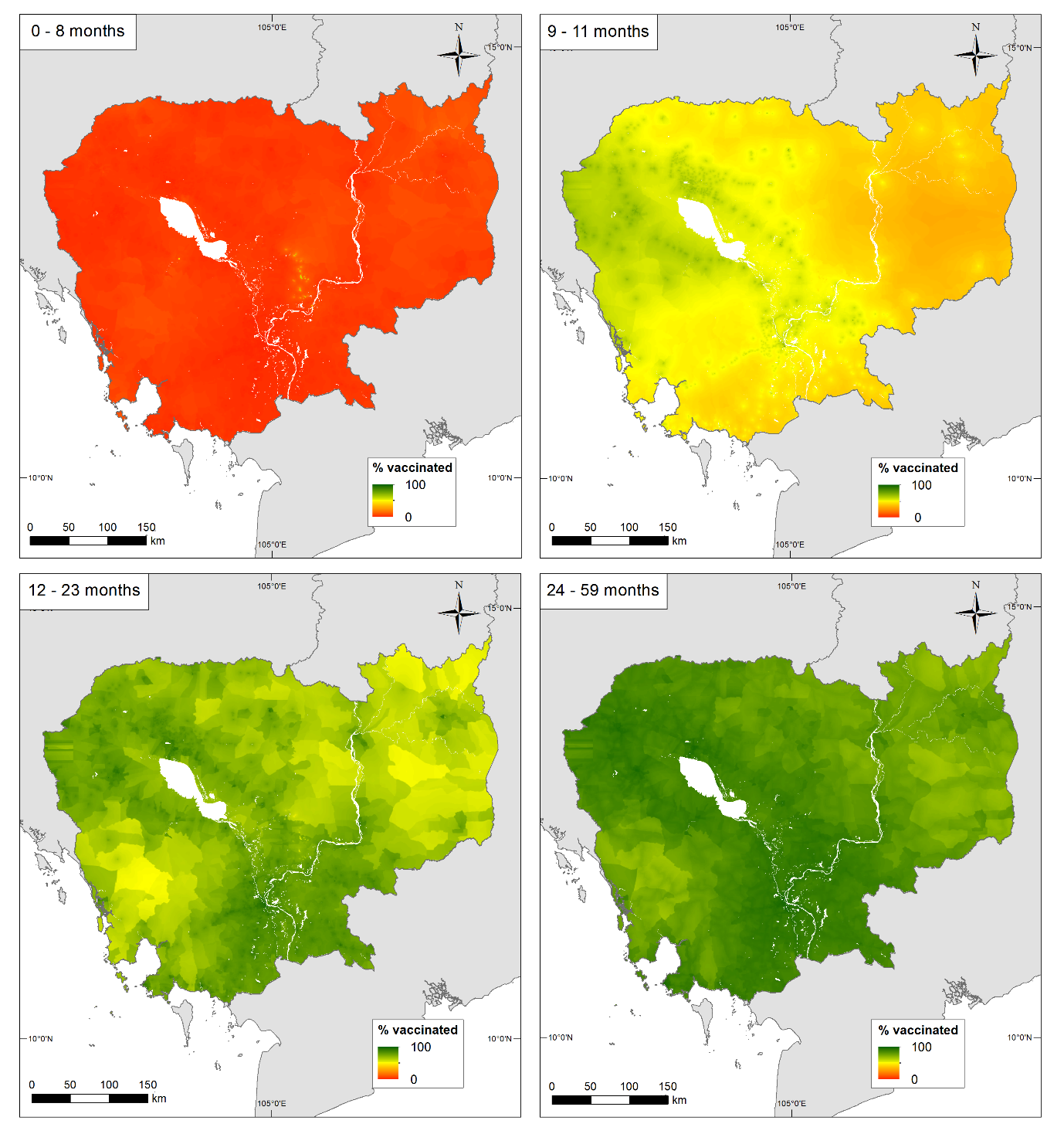


**Figure S3**. Predicted measles vaccination coverage at 1x1km for Cambodia 2013 for children (a) 0-8 months old; (b) 9-11 months old; (c) 12-23 months old and (d) 24-59 months old.


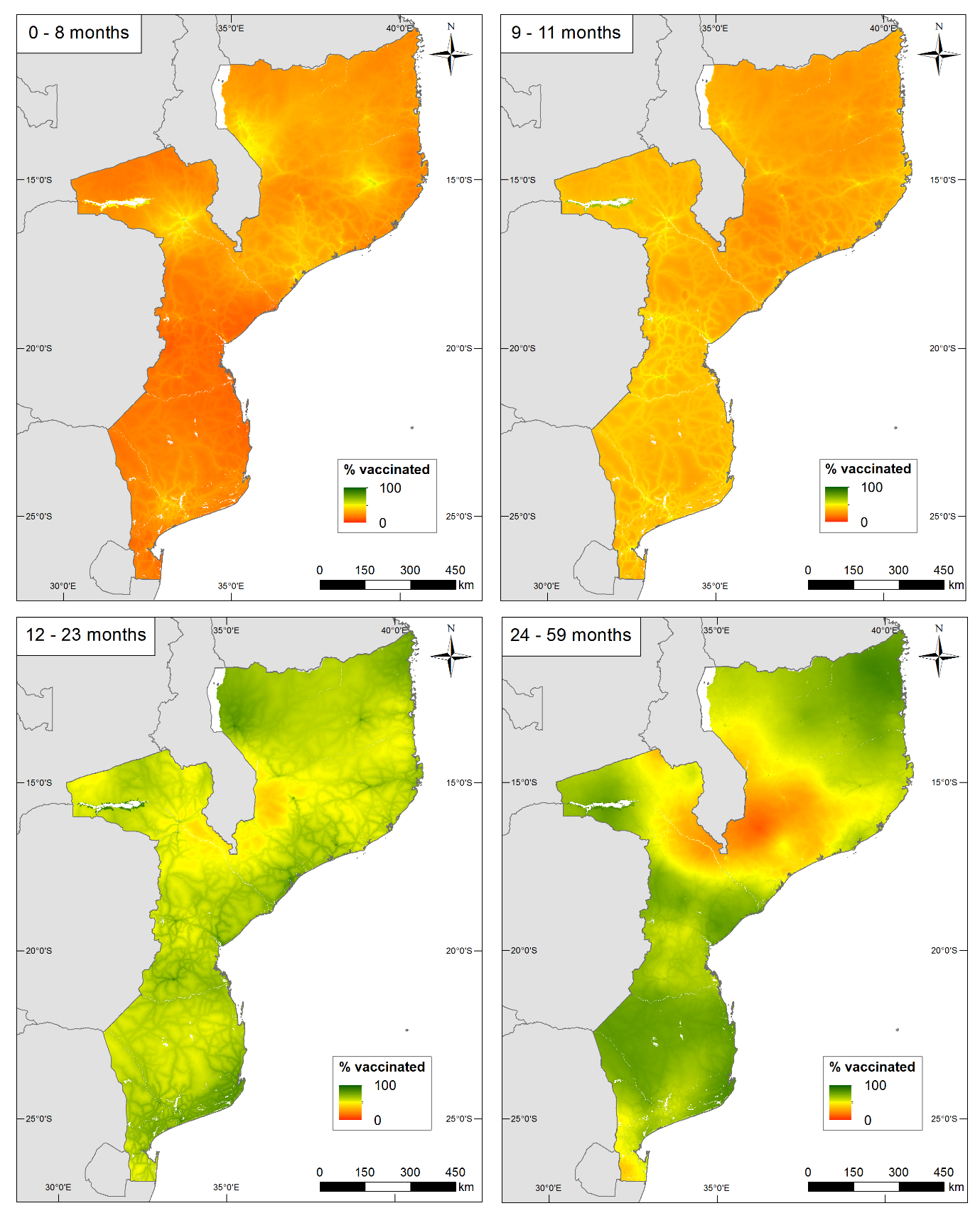


**Figure S4**. Predicted measles vaccination coverage at 1x1km for Mozambique 2011 for children (a) 0-8 months old; (b) 9-11 months old; (c) 12-23 months old and (d) 24-59 months old.


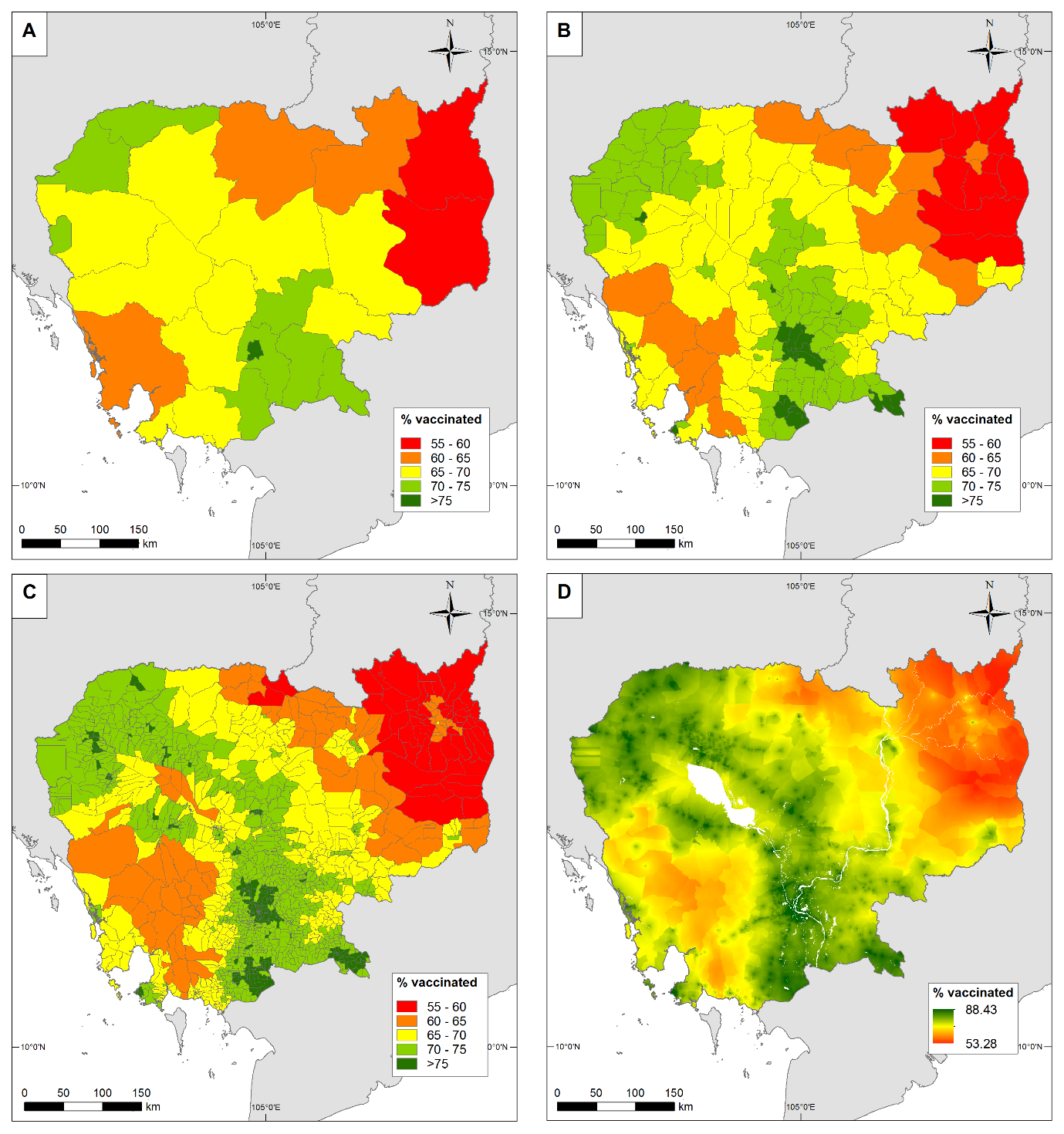


**Figure S5.** Predicted measles vaccination coverage summarised at different administrative unit levels, revealing increasing heterogeneity in coverage estimates in Cambodia at the (a) Province; (b) District; (c) Commune; and (d) 1x1km level.


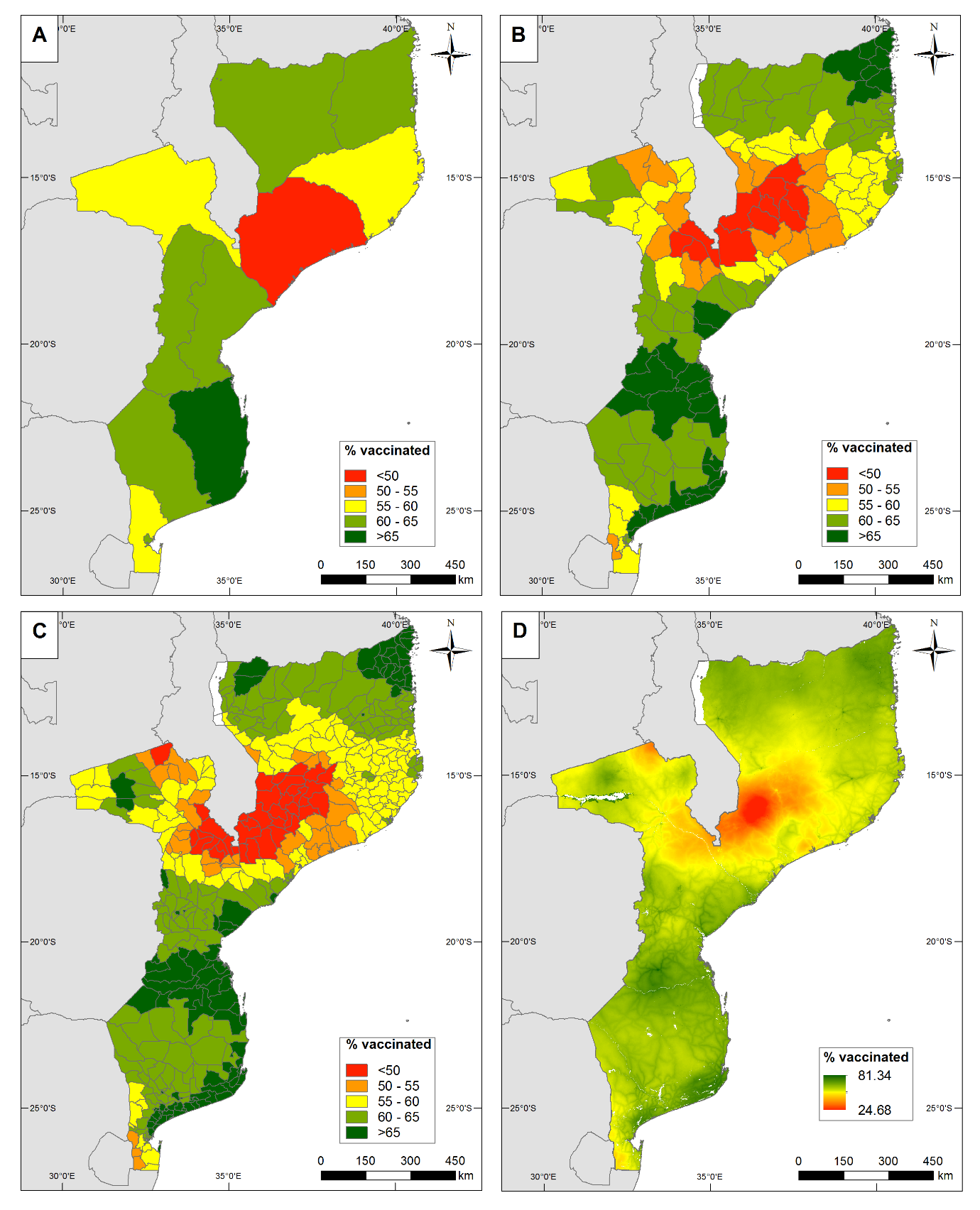


**Figure S6.** Predicted measles vaccination coverage summarised at different administrative unit levels, revealing increasing heterogeneity in coverage estimates in Mozambique at the (a) Province; (b) District; (c) Locality; and (d) 1x1km level.


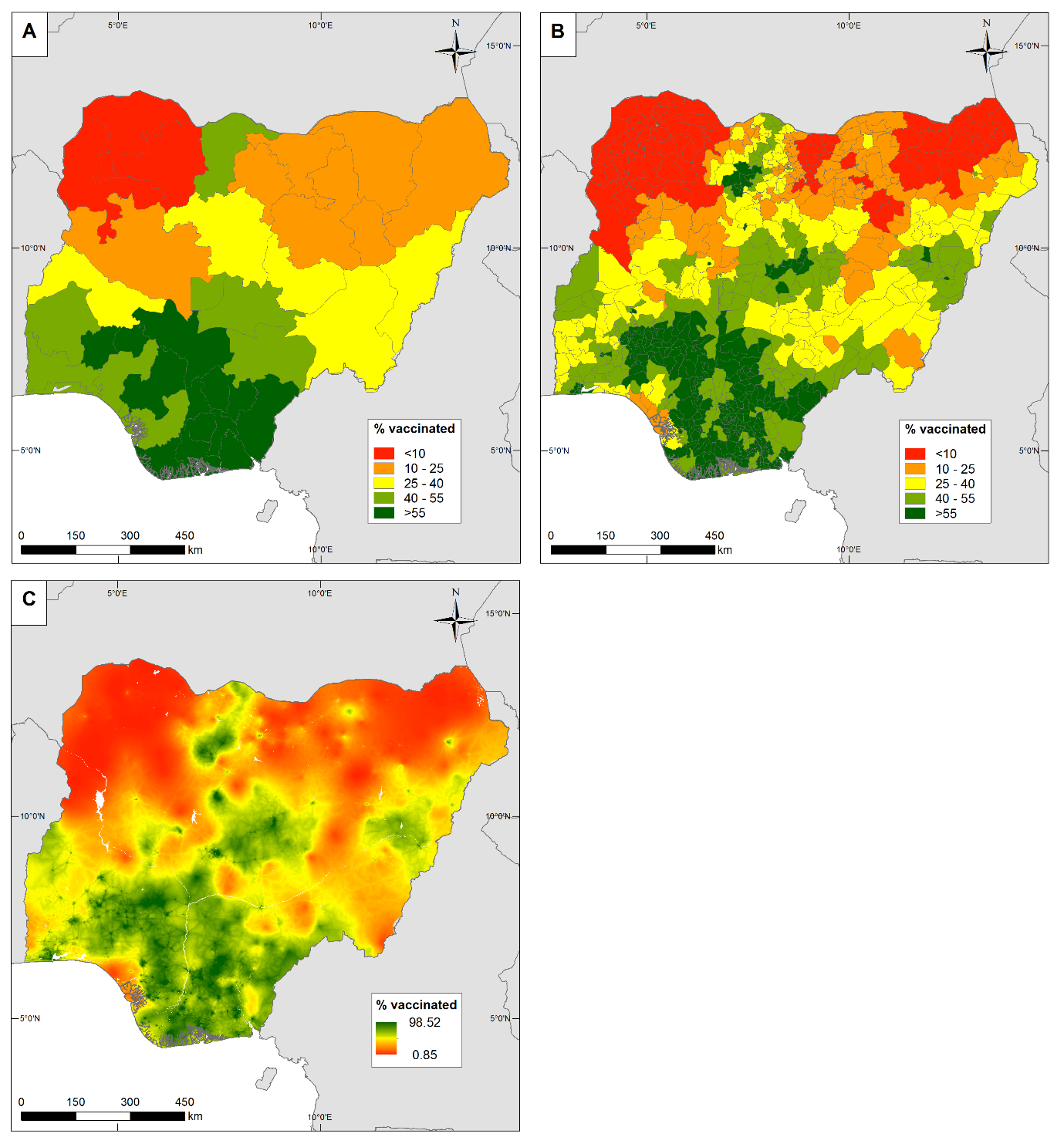


**Figure S7.** Predicted measles vaccination coverage summarised at different administrative unit levels revealing increasing heterogeneity in coverage estimates in Nigeria at the (a) State; (b) Local Government Area (LGA); and (c) 1x1km level.

***
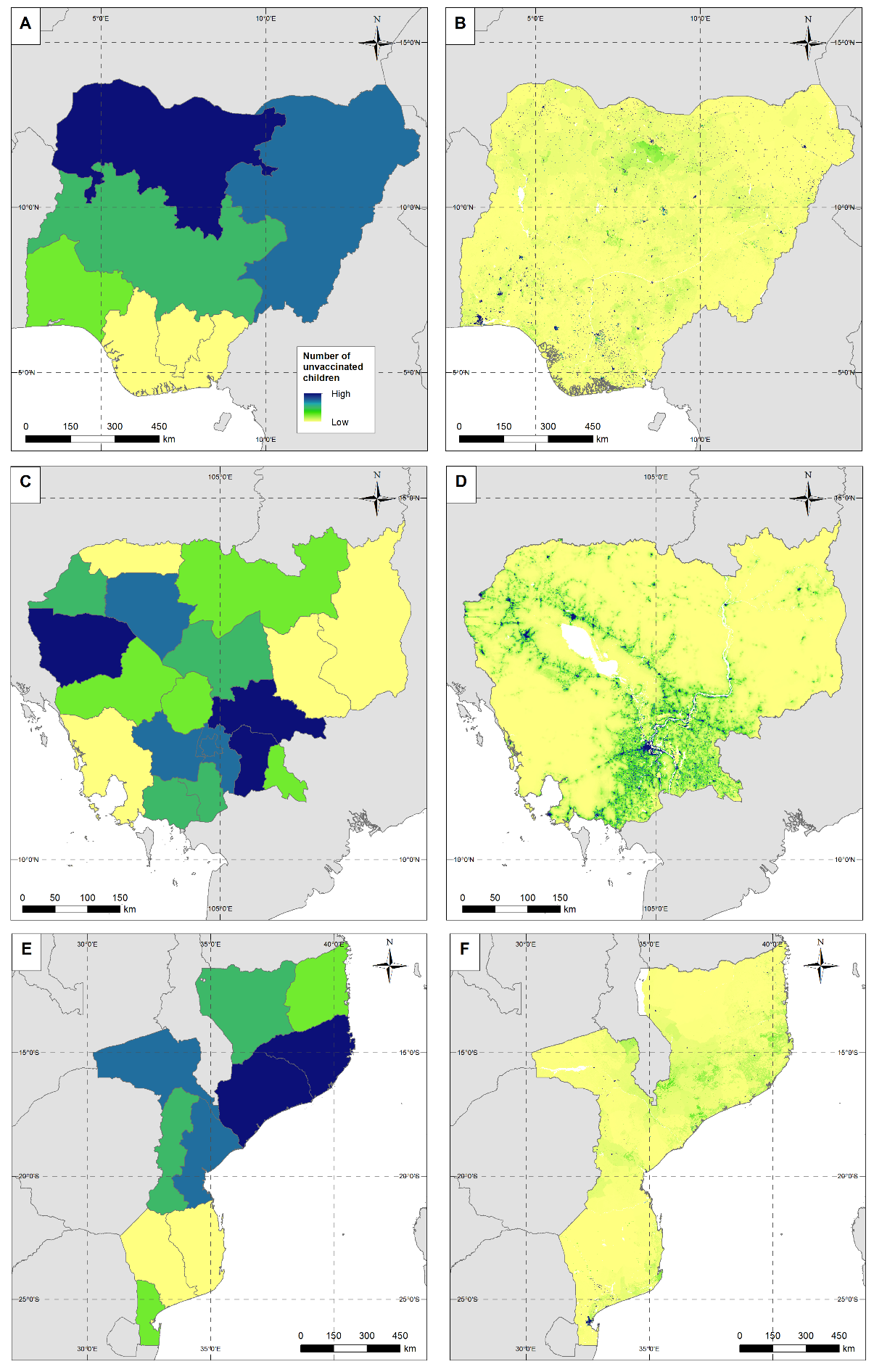
***

**Figure S8.** Estimated numbers of under 5 year old children unvaccinated against measles in Nigeria 2013 at (a) DHS region level and (b) 1x1km grid squares. The same results for Mozambique 2011 are shown in (c) and (d).

**Table S6.** National estimates of numbers of children under 5 vaccinated through using DHS region summaries and the 1x1km model predictions.

| **Country** | **Year DHS** | **UN. estimate of total pop. under 5 in year of DHS** | **DHS estimate % U5 vax.** | **DHS estimate pop U5 vax.** | **Model estimate % U5 vax.** | **Model estimate pop U5 vax.** | **Model 95% CI** |
| --- | --- | --- | --- | --- | --- | --- | --- |
| Nigeria | 2013 | 29,816,179 | 38.45 | 11,464,320 | 43.25 | 12,896,257 | 6,686,356 - 19,935,150 |
| Cambodia | 2014 | 1,763,686 | 71.86 | 1,267,385 | 73.37 | 1,294,039 | 1,188,823 - 1,399,243 |
| Mozambique | 2011 | 4,444,951 | 68.92 | 3,063,460 | 59.51 | 2,645,193 | 1,763,851 – 3,526,463 |

**Table S7.** Regional estimates of numbers of children under 5 vaccinated through using DHS region summaries and the 1x1km model predictions.

| **Nigeria regional breakdown** | | | | | | |
| --- | --- | --- | --- | --- | --- | --- |
| **DHS Region** | **DHS estimate % vax.** | **DHS estimate pop U5 vax.** | **Model estimate % vax.** | **Model estimate pop U5 vax.** | **Model 95% Lower CI pop U5 vax.** | **Model 95% Upper CI pop U5 vax.** |
| North Central | 45.68 | 2233435 | 36.88 | 1802871 | 568503 | 3448317 |
| North East | 25.51 | 1193865 | 23.68 | 1108320 | 240382 | 2590718 |
| North West | 20.15 | 1899101 | 18.31 | 1726208 | 492467 | 3740240 |
| South East | 61.69 | 1612427 | 61.23 | 1600258 | 921058 | 2178383 |
| South South | 59.55 | 2030769 | 55.45 | 1891068 | 899627 | 2788234 |
| South West | 59.68 | 2863336 | 46.29 | 2221260 | 900586 | 3702904 |

| **Cambodia regional breakdown** | | | | | | |
| --- | --- | --- | --- | --- | --- | --- |
| **DHS Region** | **DHS estimate % vax.** | **DHS estimate pop U5 vax.** | **Model estimate % vax.** | **Model estimate pop U5 vax.** | **Model 95% Lower CI pop U5 vax.** | **Model 95% Upper CI pop U5 vax.** |
| Banteay Meanchey | 76.09 | 66876 | 72.75 | 63941 | 57801 | 70080 |
| Battambang/Pailin | 71.43 | 103315 | 69.50 | 100521 | 89882 | 111159 |
| Kampong Cham | 71.87 | 158296 | 70.11 | 154425 | 140599 | 168251 |
| Kampong Chhnang | 71.87 | 44491 | 69.11 | 42782 | 39308 | 46255 |
| Kampong Speu | 71.87 | 67423 | 66.13 | 62042 | 56691 | 67394 |
| Kampong Thom | 71.87 | 59338 | 69.60 | 57461 | 52047 | 62876 |
| Kampot/Kep | 67.93 | 55048 | 66.75 | 54089 | 48136 | 60043 |
| Kandal | 73.23 | 105389 | 74.43 | 107117 | 99758 | 114476 |
| Kratie | 71.05 | 29716 | 65.48 | 27387 | 24672 | 30101 |
| Mondul Kiri/Ratanak Kiri | 63.15 | 17477 | 58.73 | 16253 | 14160 | 18347 |
| Otdar Meanchey | 71.73 | 17402 | 71.14 | 17258 | 15674 | 18841 |
| Phnom Penh | 80.00 | 164031 | 77.12 | 158121 | 149039 | 167204 |
| Preah Sihanouk/Koh Kong | 74.30 | 32321 | 65.25 | 28384 | 25016 | 31751 |
| Preah Vihear/Stung Treng | 66.87 | 24775 | 63.05 | 23358 | 20648 | 26068 |
| Prey Veng | 66.56 | 82585 | 71.09 | 88201 | 80526 | 95876 |
| Pursat | 75.46 | 39495 | 66.65 | 34887 | 31115 | 38659 |
| Siem Reap | 73.98 | 87454 | 70.14 | 82907 | 75520 | 90295 |
| Svay Rieng | 74.28 | 46881 | 72.89 | 46005 | 41987 | 50023 |
| Takeo | 76.47 | 84551 | 74.28 | 82134 | 75546 | 88722 |

| **Mozambique regional breakdown** | | | | | | |
| --- | --- | --- | --- | --- | --- | --- |
| **DHS Region** | **DHS estimate % vax.** | **DHS estimate pop U5 vax.** | **Model estimate % vax.** | **Model estimate pop U5 vax.** | **Model 95% Lower CI pop U5 vax.** | **Model 95% Upper CI pop U5 vax.** |
| Niassa | 65.92 | 181917 | 61.56 | 169893 | 115232 | 224553 |
| Cabo Delgado | 72.18 | 242248 | 64.14 | 215272 | 148968 | 281577 |
| Nampula | 69.89 | 602737 | 57.18 | 493129 | 308745 | 677513 |
| Zambezia | 53.87 | 449714 | 48.48 | 404734 | 226577 | 582890 |
| Tete | 65.83 | 274938 | 56.81 | 237234 | 145822 | 328647 |
| Manica | 70.24 | 228714 | 61.50 | 200258 | 134095 | 266420 |
| Sofala | 72.32 | 258717 | 61.71 | 220742 | 148819 | 292665 |
| Inhambane | 75.29 | 202730 | 65.70 | 176914 | 124780 | 229049 |
| Gaza | 74.35 | 154579 | 63.36 | 131728 | 88196 | 175259 |
| Maputo | 71.19 | 224902 | 59.50 | 187951 | 120984 | 254918 |
| Maputo City | 72.76 | 176096 | 66.05 | 159856 | 118249 | 201463 |

| **Sum total for all regions per country** | | | | |
| --- | --- | --- | --- | --- |
| **Country** | **Year DHS** | **DHS estimate pop U5 vax.** | **Model estimate pop U5 vax.** | **Model 95% CI** |
| Nigeria | 2013 | 11,832,932 | 10,349,985 | 4,022,624– 18,448,796 |
| Cambodia | 2014 | 1,286,865 | 1,247,273 | 1,138,125 – 1,356,422 |
| Mozambique | 2011 | 2,997,290 | 2,597,712 | 1,680,468 - 3,514,956 |

**References**

1. Perez-Haydrich C, Warren JL, Burgert CR, Emch ME. Guidelines on the use of DHS GPS data. Calverton, Maryland, USA: ICF International; 2013.

2. Fox J, Monette G. Generalized Collinearity Diagnostics. Journal of the American Statistical Association. 1992;87(417):178-83.

3. Hocking RR. A Biometrics Invited Paper. The Analysis and Selection of Variables in Linear Regression. Biometrics. 1976;32(1):1-49.

4. Banerjee S, Carlin BP, Gelfand AE. Hierarchical Modeling and Analysis for Spatial Data, Second Edition: Taylor & Francis; 2014.

5. Finley AO, Banerjee S, Gelfand AE. spBayes for Large Univariate and Multivariate Point-Referenced Spatio-Temporal Data Models. Journal of Statistical Software. 2015;1(13).

6. R Core Team. R: A Language and Environment for Statistical Computing. Vienna, Austria: R Foundation for Statistical Computing; 2017.

7. Brown LD, Cai TT, DasGupta A. Interval Estimation for a Binomial Proportion. Statistical Science. 2001;16(2):101-17.
